# Journeys, Journey Conditions, and Welfare Assessment of Broken (Handled) Horses on Arrival at Italian Slaughterhouses

**DOI:** 10.3390/ani12223122

**Published:** 2022-11-12

**Authors:** Martina Felici, Leonardo Nanni Costa, Martina Zappaterra, Giancarlo Bozzo, Pietro Di Pinto, Michela Minero, Barbara Padalino

**Affiliations:** 1 Department of Agricultural and Food Sciences, University of Bologna, 40127 Bologna, Italy; 2 Department of Veterinary Medicine, University of Bari, 70010 Valenzano, Italy; 3 ASL BA—Local Health Authority Veterinary Service, 70126 Bari, Italy; 4 Department of Veterinary Medicine and Animal Science, University of Milan, 20122 Milan, Italy

**Keywords:** ABMs, EBMs, horse, commercial transport, routes, EC 1/2005, journey conditions, welfare

## Abstract

**Simple Summary:**

Many horses are transported each year to slaughterhouses worldwide and are exposed to journey conditions that can be hazardous and detrimental to their welfare. In this study, hypothesizing that journey conditions would affect the welfare of broken/handled horses traveling toward slaughterhouses, the journey conditions and welfare status of horses on arrival at two slaughterhouses in Italy were described. Injuries (151/613 horses, 24.6%), reluctance to unload (136/613, 22.2%), and nasal (71/612, 11.6%) and lacrimal (62/613, 10.1%) discharges were the most common welfare problems, while journey duration, unloading duration, stopping at control posts, vehicle changes, traveling tied, poor driving experience, improper handling (i.e., beating with sticks), season, arrival temperature, and horse characteristics (i.e., age, BCS, long coat) were their risk factors (i.e., hazards). Our findings could be useful to provide evidence for the policymakers to implement current regulations on the protection of the welfare of horses during transport.

**Abstract:**

During horse transportation, the journey conditions are considered a welfare risk. This study aimed to document journeys, journey conditions, and welfare status of handled horses on arrival at two different slaughterhouses in Northern and Southern Italy, to find possible associations between journey conditions and welfare problems. The welfare status of 613 draft-breed and light-breed horses from 32 different journeys was evaluated on arrival at the slaughterhouses with a standardized protocol, using animal-based (ABMs) and environmental-based (EBMs) measures. The drivers’ skills and vehicle characteristics were found to be mostly compliant with EC 1/2005. The horses traveled in single bays, 90° to the direction of travel for an average journey duration of 26.5 ± 14 h. On arrival at the slaughterhouses, the horses were unloaded by handlers, via halter and rope. The prevalence of reluctance to unload, injuries, nasal, and lacrimal discharge was 22.2%, 24.6%, 11.6%, and 10%, respectively. Journey duration, unloading duration, vehicle changes, long stops, handlers/drivers’ skills, temperature, season, and horse individual characteristics were associated with horses’ welfare and health status (all *p* < 0.05). Our study confirms the hypothesis that appropriate journey conditions are of crucial importance to safeguard the welfare of broken/handled horses transported over long distances for slaughter.

## 1. Introduction

Slaughter is a key reason why horses are often transported, together with competition, breeding, leisure, and sale [1]. According to a recent estimation, 8% of the global horse population (5 million out of 59 million total) are slaughtered annually worldwide [2]. Some horses are bred for meat production, but others are slaughtered because of equine industry wastage. The latter animals frequently suffer from physical problems, even chronic ones, such as lameness, exercise-related injuries, and musculoskeletal traumas [2]. Pre-existing traumas and chronic problems represent a welfare concern since horses moved for meat production often travel long distances to reach the countries where they are slaughtered [3]. All horse transportation, but in particular commercial transport, is a complex event, constituted of many phases—preparation, loading, transit stage, unloading, and staying at lairage [2]. Each of these phases may affect horse welfare because it is characterized by different stressors [1], named also hazards [4].

The transport-related hazards may have different origins. They can be human-related hazards, such as lack of experience in horse handling and poor driver skills; management-related hazards, such as inappropriate or prohibited transport and husbandry practices; vehicle-related, such as inadequate ramps or ventilation systems; but they may also concern the animals, such as lack of training in loading and traveling [4]. In the literature, the most common hazards/journey conditions considered risk factors for the welfare of horses traveling by road are the following: journey duration [5]; direction of travel [6]; the restriction of the head movements [7]; stocking density [2,8]; external and vehicle’s temperature and humidity [9]; season [7]; water and food availability [10]; experience of handlers [11]. However, monitoring the hazards’ effects on animals during transit is difficult [12]. For this reason, several welfare protocols containing animal-based (ABMs) and environmental-based (EBMs) measures have been developed in recent years, to assess horses’ welfare at unloading and lairage [3,10,13,14].

On arrival at the slaughterhouse, several animal-based welfare indicators can be assessed [13]. For example, the presence of injuries can be validated and associated with prolonged journey duration [3,5], the inexperience of animal handlers [11], and a high stocking density [2,8]. The presence of nasal discharge was considered a feasible measure, and its presence is associated with prolonged journey durations [7], high stocking densities [2,8], and elevated horses’ head positions in transit [1,11,15]. A “head-up” posture in horses during transit is linked to being the cause of the prevalence of 23% and 12% in ocular and nasal discharge on a total of 2648 horses [14]. Finally, signs of sweating could be easily assessed on arrival and were associated with heat stress and its hazards, namely season, water availability, and vehicle high effective temperature [8,10]. It is therefore reasonable to assume that the presence of a particular ABM on arrival reflects the presence of one or more hazards (i.e., journey conditions) during the journey [4,12].

Worldwide, journey conditions are regulated by specific codes, such as “European Regulation 1/2005” [16], “Australian Animal Welfare Standards and Guidelines” [17], “Code of practice for the care and handling of Equines” [18], “Code of welfare: Transport within New Zealand” [19]. However, the requirements of these different regulations do not always match, and some criteria remain open to interpretation or have been considered inappropriate [20]. For example, European Council Regulation (EC) 1/2005 sets a minimum space allowance of 1.75 m^2^ per horse, even if adult horses may have different sizes and shapes [2,12,16]. For this reason, the European Food Safety Authority expressed the need to use stocking density instead of space allowance or to a space of at least 20 cm around the horse traveling in a single bay [12]. Moreover, those codes must be properly enforced. A recent study on road inspections conducted in Europe reported that overcrowding, missing scheduled stops, lack of fitness for travel, and improper tying were the most common animal welfare infringements while lacking or improper drinking and ventilation systems were the most common vehicle-related infringements [21]. For these reasons the EC 1/2005 is currently under revision, and studies providing evidence on how to improve it are needed [12].

Hypothesizing that journey conditions would affect the welfare of handled horses traveling toward slaughterhouses, the study aimed to describe their journeys and journey conditions, document their welfare status on arrival using a standardized protocol [22], and investigate possible associations between journey conditions (i.e., hazards) and welfare status (i.e., welfare consequences).

## 2. Materials and Methods

### 2.1. Sample Size Calculation

In 2021, 29,937 horses were slaughtered in Italy. Of these, 30.75% (about 9206) were slaughtered in Apulia (Southern Italy), and 17.49% (about 5236) were slaughtered in Emilia-Romagna (Northern Italy) [23], which are the two regions considered in this study. Using Statulator^®^ [24], a power analysis was performed to determine the sample size representative of a target population of slaughtered horses estimated at 14,442. The final number of subjects to include in the present one-year cross-sectional study was estimated assuming an expected proportion of welfare issues of 18% (proportion of horses with poor welfare found by Miranda-de la Lama et al. (2020) [14]), with 5% absolute precision and 99.9% confidence interval (CI). The minimum sample size obtained was 613 horses, with a 99.9% confidence interval.

### 2.2. Experimental Protocol

The study was conducted between June 2021 and May 2022, obtaining data from 32 commercial journeys, for a total of 624 sport and draft-breed horses transported in 8 different vehicles (some vehicles traveled multiple times, namely vehicles ID 1, 2, and 4) to two slaughterhouses, one in the Southern (Apulia) and one in the Northern Italy (Emilia Romagna). All journeys were approved by the Competent Authorities before departure, checking for journey plans, horses’ fitness for transport, and vehicle compliance with current regulations. On arrival at slaughterhouses in Italy, the welfare status of the horses was assessed following the protocol described by Zappaterra et al. (2022) [22].

The assessment was performed three times, filling in a specific checklist, during unloading at the slaughterhouse, 30 min, and 24 h after unloading while the horses were kept in the lairage (Tables S1–S3). The protocol, modified from Messori et al. (2016) [13], included animal-based measures (ABMs) and environmental-based measures (EBMs) recorded at three levels, namely driver, vehicle and animal, and the collection of information from official transport documents (i.e., Trade Control and Expert System—TRACES, Journey Log, International Consignment Notes—CMR, passports of the horses). Each horse’s identity number (ID) (i.e., microchip number), country of origin, sex, age, and breed (categorized as sport and draft-breed horses) were obtained from the passport, and the ID was double-checked using a microchip reader. All the other ABMs were obtained by direct visual observation.

The assessment completed during the unloading (see Table S1 for the checklist) started as the ramp was opened and ended with the unloading of the last horse. In that checklist, there were 11 EBMs, of which 5 concerned the human–horse interactions and 12 ABMs. The EBMs were external environmental temperature and humidity, date and time of arrival, duration of unloading, and the total number of horses per load. In addition, the presence of handlers performing prohibited practices, moving horses in an arousing/noisy manner, inducing fear, or hitting them was recorded. Mutually, the presence of handlers showing a positive attitude (i.e., moving calmly, talking soothingly), and moving horses properly was also recorded. The ABMs recorded were the number of dead on arrival (DOA), the number of non-ambulatory, reluctant to move/unload, or lame horses (detected in a mutually exclusive way; non-ambulatory = unable to assume and maintain a standing position; lame horses = unable to ambulate normally; reluctant to move/unload = unwillingness to move/unload), number of animals that showed difficulties in maintaining the balance (slipping/losing balance or falling) as well as those which assumed fast gaits getting out of the vehicle (galloping or jumping). Body Condition Score (BCS) was also assessed, with only the number of animals deemed to have a score between 0–1 (emaciated) or equal to 5 (obese) (i.e., welfare issues) noted down. Lastly, the number of horses showing signs of sweating, injuries, considered exhausted, or coughing was recorded.

The assessment performed 30 min after unloading consisted of an individual inspection of each horse (see Table S2 for the checklist) while it was kept at the lairage facilities. Four EBMs and 17 ABMs were included in the assessment. The EBMs were the date and hour of the assessment, hours of travel, and the location of where the horse was kept. The ABMs were presence/absence of halter, BCS, estimated weight, demeanor, heart, and respiratory rate, sweating signs, coat length, lameness, gastroenteric (abnormal feces, clinical signs of colic), and respiratory (nasal discharge, type of nasal discharge, cough) clinical signs, and any other discharge. The number of cuts and injuries was counted, and the affected body part was also noted (e.g., tail). Any other clinical signs were also described. The horse’s ID was also rechecked using a microchip scanner.

Once the animals had been checked, the assessor interviewed the drivers and inspected the vehicles (see Table S3 for the checklist). The assessor asked the driver for his/her age, years of experience, and whether he/she possessed the certificate of competence (similar to a license, issued by the Competent Authorities to the drivers/transporters following the attendance of a specific course, in accordance with EC 1/2005 [16]). During vehicle inspection, 26 EBMs were collected: dimensions (m^2^), presence of mandatory/non-mandatory monitoring/controlling systems (forced ventilation, temperature monitoring, and cameras), drinking method (total and working number and type of drinkers, and water tank filling), lighting systems (presence/absence of internal and external lights), and characteristics of the ramp (height, covering, flooring type and condition, side gates, and compliance of the slope), of the deck (height), and of vehicle’s floor (if there was a covering and of which type). The structure (presence of adjacent stalls) and dimension (width and length) of the single bays/compartments were recorded. Any potentially injurious structural protrusions or hazards on the vehicle were recorded if detected (presence/absence of slot/steps, blockage zone on the ramp, and sharp edges). Traveling position (direction of travel and presence/absence of horses in groups), tethering method, and any signs of diarrhea inside the vehicle were noted down. Moreover, the documentation concerning TRACES, Journey Log, horses’ passports, and CMR was recorded.

Twenty-four hours after arrival, the assessor re-checked the horses individually (see Table S4 for the checklist) while they were kept in the lairage facilities. The same initial 5 EBMs collected in the checklist 30 min after unloading were collected again. Then, 6 environmental/paddock/stabling characteristics were checked, namely paddock temperature and humidity, number of horses per paddock/box, paddock’s exposure to the sun, level of cleanliness, and freedom of movement for the horses. Moreover, 13 of the 15 ABMs collected in the checklist 30 min after unloading were re-checked (for further details, see Table S4). Finally, each horse was assessed using the Broken/Unbroken Test [25]. The BUT was composed of two behavioral tests, namely the Approaching and Handling tests. During the Approaching test, the slaughterhouse caretaker entered the lairage facility, slowly approached the horse, and tried to halter the animal. Only if this was successful, the caretaker tried to make the horse move three steps forward and three backward (handling test). During the tests, the assessor observed and scored the horse behavior as described in Menchetti et al. [25], from 0 to 4, identifying as “unbroken” the horses scoring < 2 and as “broken” the horses scoring ≥ 2. In the present study, it was not possible to perform the BUT test on most of the horses that arrived at the slaughterhouse in Northern Italy, because the animals were slaughtered before 24 h of rest at the lairage. However, all of these horses had halters and ropes during transport, which had been used during unloading to guide the horses towards the lairage pens. For this reason, they were considered broken horses, and only the horses considered broken were included in this dataset (n = 613). In this paper, according to EFSA terminology, we decided to name “broken” horses as “handled”.

The protocol was applied by a freelance practitioner veterinarian (P.D.P.) in the Southern Italy slaughterhouse, and by the researchers of the University of Bologna (B.P., M.Z.) in the Northern Italy slaughterhouse. The veterinarian and the researcher (M.Z.) were trained with images and video recordings by the senior scientist (B.P.) to apply the protocol. To test interobserver agreement, the welfare protocol was applied individually by the veterinarian (P.D.P.), the senior scientist (B.P.), and the researcher (M.Z.) on videos of horses collected previously on other journeys. An excellent interobserver agreement between the veterinarian and the researchers was noticed using Cohen’s k (k > 0.70). However, at the Northern Italy slaughterhouse, the unloading phase was also recorded through a video camera (HDR-CX115E, Sony, China) to double-check the measures in case of doubts. Due to the impossibility of conducting accurate auscultation of each horse, the heart rate was never recorded. Deck and ramp height were checked for compliance with EC 1/2005 regulation [16], but because of technical problems, ramp height was not measured, and deck height was measured only for two vehicles.

### 2.3. Data Handling and Statistical Analysis

All information collected during the unloading, 30 min, and 24 h after arrival were compared with those reported on TRACES, Journey Logs, passport, and CMR. If some information was missing from the checklists, they were retrieved from the other documentation. In addition, Journey Logs and TRACES were used to identify whether the animals were transported on a single vehicle or experienced a vehicle change during the journey, thus being unloaded and loaded onto a second vehicle (Table 1).

#### 2.3.1. Data Handling and Statistical Analysis

The data collected for each journey was inputted and organized into Excel data sheets, one for the journeys and one for the individual horses.

In the journey sheet, each row was a journey, and columns reported driver variables; vehicle-related EBMs; ABMs; and information retrieved from the official documentation. For each journey, the number of horses showing health issues/behavioral events at unloading was calculated, and it was expressed as the percentage of horses per vehicle ((number of horses showing health issues or behavioral events/total horse number) × 100).

In the horses’ sheet, each row was a horse and each column reported the ABMs recorded at 30 min and 24 h after arrival, the EBMs recorded at the lairage pens and the BUT score, and the vehicles and journey characteristics. For several horses transported to the slaughterhouse in Northern Italy, the assessment undertaken at 24 h after arrival was not able to be conducted due to those animals being slaughtered approximately 15 h after their arrival. For this reason, the information collected at 30 min after arrival was combined with those gathered at 24 h meaning that: for horses assessed both at 30 min and 24 h after arrival, the detected health issues were constant over time and thus the values of the ABMs as recorded at 24 h were retained; for horses that were slaughtered before the 24 h assessment was conducted, the ABMs collected at 30 min after arrival were retained. From this point on, we will not refer to a welfare status detected at two times (30 min and 24 h), but to a single “welfare status after unloading”. The injured horses were also categorized as to the location of the cuts or bruises (i.e., tail, head/neck, trunk, and limbs). However, the presence of ruffled tails was annotated for horses traveling on 15 out of 17 total journeys, as this assessment was added a second time to the checklist.

#### 2.3.2. Descriptive Statistics and Regression Models

In order to carry out the statistical analyses, the whole set of data was then organized into continuous, categorical, or dichotomous variables (Table S5). The descriptive statistics of the dataset were performed using Statulator^®^ [26]. Continuous variables were expressed as the minimum (Min), maximum (Max), and mean values ± standard deviations (S.D.). Categorical and dichotomous variables were represented as percentages and counts. Based on the results of the descriptive statistics, only the variables being present in at least 5% of the individuals were further considered for determining the possible associations using the regression models. Before performing the regression models, collinearity between categorical and continuous variables was tested using Kendall’s tau statistic with the *cor.test* function in the R environment or *glm* function for dichotomous variables [27]. The factors ‘duration from journey log’, ‘duration from TRACES’, and ‘hours in transit’ were collinear, and only ‘duration from journey log’ was retained in the regression models, to avoid confounder factors. Similarly, the factors ‘departure country’ and ‘country of origin’ were collinear between themselves and also with ’journey durations from TRACES’ and therefore were not included in the regression models. In addition, the factors ‘vehicle dimension’, ‘number of horses transported’, ‘load weight from CMR’, ‘load weight from TRACES’, ‘space allowance’, and ‘stocking density’ were collinear, and only ‘stocking density’ was retained among the factors used in the regression models. The factor ‘The handler performs prohibited practices’ was collinear with the factors ‘The handler moves the animals in an arousing manner, inducing fear’, ‘The handler hits the animals’, and ‘The handler moves the animals properly’; consequently, only the latter was considered in the regression models. Furthermore, the factors ‘presence of forced ventilation’, ‘internal temperature monitoring system’, ‘lighting system’, and ‘presence of cameras’ were excluded as possible factors, as it was not possible to check whether these facilities were actually used during the journey. Moreover, all the factors showing no variability in the dataset were removed as possible factors (e.g., ‘certificate of competence’; ‘vehicle covering’; ‘appropriate ropes’; ‘appropriate deck’; ‘direction of travel’; ‘appropriate ramp flooring conditions’; ‘blockage zones (holes or physical obstacles) on the ramp’). Table 2 shows the final list of factors retained for the univariable regression models.

In order to identify associations between welfare issues (such as health parameters observed after unloading) and the factors included in Table 2, multivariable regression analyses were performed. The list of outcome variables is reported in Table 3.

The multivariable regression analyses were performed as follows. First, the associations between factors and outcomes were tested using univariable logistic regression models. Then, for each outcome, the factors that showed a *p* < 0.100 in univariable models were considered for inclusion in the stepwise multiple regression. A stepwise backward elimination procedure was conducted for each dependent outcome variable to test the combined effect of the factors. In the backward multivariable regression analysis, the factors were removed one at a time until all of them in the final model had a *p* < 0.100 or the final model had a lower Aikake Information Criterion (AIC) value than the other possible models. Only for the outcome variable of lacrimal discharge, the association with the presence of bilateral or unilateral nasal discharge was also tested with a univariable regression model, and based on the results, nasal discharge was included among the factors tested during stepwise backward selection. The final multivariable models resulting from the stepwise backward selection are presented with the model *p*-value and AIC, and the effects of the factors were reported as estimates, odds ratio (OR), 95% Confidence Interval (CI), and *p*-values. The model *p*-value was estimated with Wald test statistics while the *p*-values of the estimated effects were obtained from the outputs of the Generalized Linear Models (GLMs) and applying type 3 Anova with F statistics to the GLM estimates. The significance was set at *p* < 0.05, and a trend towards significance was considered for 0.05 < *p* < 0.1. The scripts used to perform the univariate logistic and stepwise multiple regressions were a combination of functions in the packages *nlme*, *lsmeans*, *lme4*, and *car* in R environment [27].

## 3. Results

### 3.1. Description of the Routes and Country of Origin

**Figure 1:**
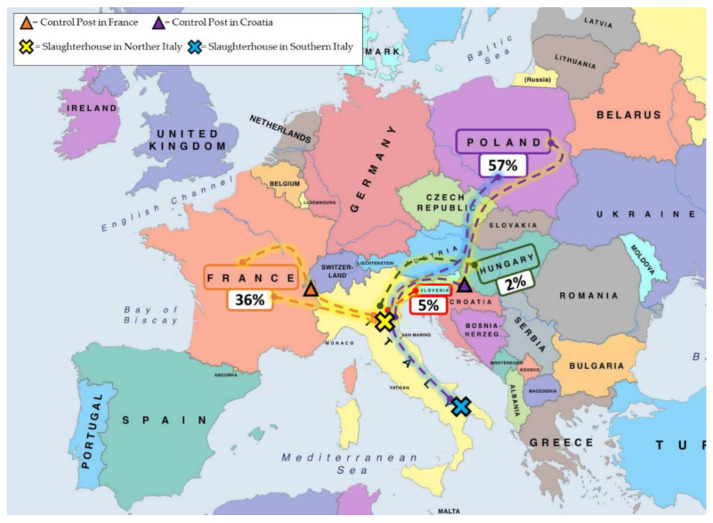
Departure countries of the 624 horses travelling to two Italian slaughterhouses between 2021 June 2021 and April 2022. In the boxes is the percentage of horses departing from each country.

The horses’ countries of departure were Poland (17/32 journeys, 53%; 358/624 horses, 57%), France (12/32 journeys, 38%; 224/624 horses, 36%), Slovenia (2/32 journeys, 6%; 32/624 horses, 5%), and Hungary (1/32 journeys, 3%; 10/624 horses, 2%) (Figure 1).

Poland (350/624 horses; 56%), France (220/624 horses; 35%), Slovenia (29/624 horses; 5%), Hungary (11/624 horses; 2%), Croatia (3/624 horses; 0.5%), Czech Republic (3/624 horses; 0.3%), Ireland (2/624 horses; 0.3%), United Kingdom (2/624 horses; 0.2%), Germany (1/624 horses; 0.2%), and Holland (1/624 horses; 0.2%) were the horses’ countries of origin. All journey details are reported in Table 4.

#### 3.1.1. Routes from Poland to Southern Italy

Fifteen journeys (15/32, 47%; 320/624 horses, 51%) departed on different dates from the same assembly center in Święck Wielki (Wysokie Mazowieckie, Poland). All these journeys had the same route. The horses traveled 6 h 15 min ± 45 min (min = 4 h; max = 7 h 25 min) before the short stop (1 h) in the Czech Republic. Here, the horses were watered, without being unloaded from the vehicle. The journeys continued for 4 h 45 min ± 45 min (min = 3 h 55 min; max = 6 h 30 min) towards a Control Post (CP) in Croatia (Hrašćina, Croatia). Here, the animals were unloaded, watered and fed, and rested for 12 h. Subsequently, the horses traveled for 7 h 45 ± 45 min (min = 5 h 30 min; max = 8 h 30 min) before the short stop of 1 h in Northern Italy, where the animals were watered on board. Finally, the journeys lasted 8 h 30 min ± 30 min (min = 7 h; max = 8 h 30 min) toward the arrival slaughterhouse. The vehicle and the driver were always the same.

#### 3.1.2. Routes from Poland to Northern Italy

Two journeys departed from an Assembly Center in Zaborów Drugi (Tomaszów Mazowieckie, Poland) (2/32 journeys, 6%; 38/624 horses; 6%). The journeys lasted on average 5 h ± 3 h (min = 2 h; max = 8 h). During the journey, one short stop of 1 h was performed, respectively, in Poland (1/32 journeys, 3%; 19/624 horses, 3%) or Hungary (1/32 journeys, 3%; 19/624 horses, 3%) without unloading the horses. After the short stop, the horses traveled for 5 h 45 min ± 3 h 45 min (min = 3 h 05 min; max = 8 h 35 min) and stopped in Hungary for 1 h. The journeys continued for 9 h ± 1 h 30 min (min = 8 h 05 min; max = 9 h 55 min) toward the slaughterhouse.

#### 3.1.3. Routes from France to Northern Italy

Ten journeys departed from an Assembly Center in Thoigné (10/32 journeys, 31%; 190/624 horses, 30%) (Sarthe department, Loire countries, France).

Two journeys (2/32 journeys, 6%; 38/624 horses, 6%) lasted on average 4 h 15 min (min = 4 h 10 min; max = 4 h 20 min) before a short stop (30 min) in France. After that, the horses traveled 2 h 30 min ± 15 min (min = 2 h 20 min; max = 2 h 45 min) towards a parking area in France, where they were watered, fed, and rested for 9 h without being unloaded. The journeys continued for 4 h 45 min ± 15 min (min = 4 h 35 min; max = 4 h 50 min), with a short stop of 45 min in France. Finally, the horses traveled 4 h 15 min ± 30 min (min = 3 h 40 min; max = 4 h 30 min) toward their final destination.

Eight journeys (8/32 journeys, 25%; 152/624 horses, 24%) lasted 3 h 30 min ± 30 min (min = 2 h 45 min; max = 4 h) before a short stop (40 min ± 10 min) in France. Subsequently, the horse traveled for 3 h 45 min ± 30 min (min = 3 h; max = 5 h) towards a Control Post in France (Bourg-en-Bresse). Here, the horses stayed for 2 h and were unloaded, watered, fed, and moved into a new vehicle. The journeys continued for 3 h 45 min ± 45 min (min = 2 h 15 min; max = 4 h 40 min) before stopping shortly (1 h 10 min ± 20 min) in Italy (6/32 journeys, 19%; 114/624 horses, 18%) or France (2/32 journeys, 6%; 38/624 horses, 6%). After these short stops, 5/8 journeys reached the slaughterhouse after 3 h 45 min; instead, 3/8 journeys continued for 3 h 15 min ± 45 min (min = 2 h 30 min; max = 3 h 50 min) and performed another short stop (40 min ± 10 min) in Italy, before reaching the slaughterhouse after 2 h 30 min ± 30 min (min = 2 h; max = 2 h 50 min).

Two journeys (2/32, 6%; 34/624 horses, 5%) departed from an Assembly Center in Arlay (Giura department, Bourgogne-Franc County, France). These journeys lasted, on average, 5 h ± 45 min (min = 4 h 15 min; max = 5 h 25 min). During the journeys, only one short stop (45 min) was performed in Italy. The journeys continued for 4 h 45 min ± 1 h 45 min (min = 3 h 20 min; max = 6 h) to reach the arrival slaughterhouse.

#### 3.1.4. Routes from Slovenia to Northern Italy

Two journeys (2/32, 6%; 32/624 horses; 5%) departed from Šentjernej (Lower Carniola, Slovenia). There were no stops on these routes, as journeys lasted less than 8 h (duration from TRACES: 5 h), and the drivers did not have to compile the Journey Log. Although the exact departure time remains unknown, over a distance of 462 km, retrieved from Google Maps, assuming an average vehicle speed of 80 km/h, a journey duration of around 6–7 h was estimated.

#### 3.1.5. Routes from Hungary to Northern Italy

Only one journey (1/32 journeys, 3%; 10/624 horses, 2%) departed from Söjtör, Hungary (Zala, Western Transdanubia). This journey reported on Journey Log a total duration of 9 h 45 min, and two short stop locations, respectively, in Hungary and Slovenia. However, the Journey Log was filled in an unclear way, and it was impossible to understand whether the two locations were stops or just transit points.

#### 3.1.6. Documentation and Journey Duration

Table 5 reports the descriptive statistics for the journey duration declared on TRACES, the actual journey duration calculated from Journey Logs, and the hours in transit. The minimum journey duration declared was 5 h, which was reported on the TRACES for the two journeys coming from Slovenia. Journey Logs were not available for these two journeys and thus it was not possible to obtain the actual journey duration or the hours in transit.

Concerning TRACES accuracy, the 15 journeys from Poland to Southern Italy had all TRACES parts accurately filled in, with a journey duration very similar to the actual duration reported on the Journey Log (average difference between the duration declared on TRACES and the actual duration from Journey Log of less than 1 h). Concerning journey breaks, all the horses transported during the 15 journeys from Poland to Southern Italy were subjected to a long stop of 12 h (15/32; 46.87%). Short stops lasted on average 77 ± 84 min and the median number of short stops per journey was 2, with 17 journeys having vehicles stopping twice (17/32 journeys; 53%), 7 journeys with vehicles stopping thrice (7/32; 22%), 3 journeys with four short stops (3/32; 9%), and 2 journeys with vehicles stopping once (2/32; 6%).

### 3.2. Description of Vehicles and Journey Conditions

Table 6 reports the descriptive statistics of the driver’s information and journey conditions. The vehicles arrived at the two slaughterhouses throughout the four seasons, with average arrival temperatures of 20.2 ± 7.2 °C, ranging from a minimum of 8 °C to a maximum of 36 °C. All drivers had a long experience in animal transport, being involved in this sector for at least 15 years, and had a competence certificate issued by competent authorities. Vehicles had an average dimension of 34.10 ± 3.66 m^2^ and transported a median number of 19 ± 3 horses, which had in most of the journeys a space allowance below 1.75 m^2^/animal (17/32, 53.1%). Stocking density varied from a minimum of 203.92 kg/m^2^ to a maximum of 384.40 kg/m^2^. Most of the vehicles were equipped with fixed bowls as drinkers (27/32 journeys; 84.4%), for a median number of 20 drinkers per vehicle. During five journeys, the vehicles were not equipped with fixed drinkers and the horses were watered with portable drinkers.

All but 11 horses were transported in individual stalls with an orientation of 90° to the direction of travel, with the head toward the right side of the vehicle. The 11 horses were one-year-aged draft-breed horses coming from Poland, born in Slovenia, and transported in three journeys in groups of two to four animals. During these three journeys, all the other horses were transported in single stalls, while in the part of the vehicle closest to the cabin a larger stall was created in which the young horses were transported in groups. The 11 horses were declared to be unhandled, and their level of tameness was confirmed by the results of the BUT test (BUT score < 2) (i.e., excluded from the rest of the analysis).

Table 7 shows the frequency of vehicles displaying the different characteristics and the relative number of handled horses transported in those journey conditions. Horses were always transported on vehicles with one deck, having an adequate height. All horses coming from France were transported loose in individual stalls (12/32 journeys, 37.5%; 223/613 horses, 36.4%), while the horses coming from Poland and other Eastern European countries were tied with a single rope in transit (20/32 journeys, 62.5%; 390/613 horses, 63.6%). Rope length was, however, considered to be adequate (i.e., more than 60 cm) by the assessor. Vehicles were always equipped with forced ventilation, while cameras for the control of animals during travel were present in three vehicles, consequently only during 13 journeys (13/32; 40.6%; 233/613, 38%). On 20 journeys (20/32, 62.5%), stallions were transported in stalls adjacent to females or other stallions. The bedding quantity in the vehicle was sufficient to cover the vehicle flooring in almost all journeys (31/32, 96.9%). Straw was used as bedding material in half of the journeys (17/31, 58.8%; 358/614 horses, 58.3%). In most of the cases, the water tank was partially empty at arrival (24/27, 88.9%). Potentially harmful openings or sharp edges inside the vehicle were never noticed. Ramp flooring was made of non-slip knurled metal with foot battens (16/32; 50%), or corrugated metal or rubber mat (16/32; 50%). Ramp side gates were present in almost all vehicles (31/32, 97%). Vehicles equipped with lighting for animals’ orientation were also equipped with lighting for loading/unloading of the animals (15/32 journeys, 46.9%), and during 9 journeys the animals experienced a vehicle change on the border between France and Italy (9/32, 28.1%). In more than half of the observed journeys, the handlers at the slaughterhouse performed prohibited practices, such as using sticks during unloading or did not move the animals properly (17/32, 53.1%).

### 3.3. Description of Unloading Conditions and Behavioral Parameters at Unloading

Unloading lasted on average 26 ± 6 min (median = 30 min), ranging from a minimum of 10 min to a maximum of 35 min. At arrival, signs of diarrhea were present inside two vehicles (2/31, 6.4%).

No DOA or exhausted horses were observed, and all horses were able to stand and walk unaided during unloading procedures. One horse was, however, in pain and limped, with an evident lesion in the right coxal tuberosity that likely occurred during transport (1/613, 0.2%).

At arrival at the slaughterhouse in Northern Italy, two horses from different journeys (2/32, 6.25%; 2/613, 0.3%) were considered not fit for transport as they were severely lame (lameness score > 3) with laminitis and a clear rotation of coffin bone, which could have not happened during the journey. Video S1 shows one of these two horses considered to be unfit for transport. The horse came from Slovenia and had dirty long hair, with signs of neglected hooves.

Except for one journey, at least one horse per journey was reluctant to move at unloading (31/32, 96.9%; 136/613, 22.2%), with an average frequency of reluctant animals per journey of 22.1 ± 13.9% (min = 1.24%; max = 52.4%, median = 20%). Overall, 50 animals (50/613, 8.2%) from 19 journeys (19/32, 59.4%) were beaten with sticks or rubber mats, with an average frequency for the 19 journeys of 12.7 ± 7.8% (min = 4.5%; max = 31.6%; median = 10.5%). At unloading, 83 horses (83/613, 13.6%) from 17 journeys (17/32, 53.1%) galloped or jumped while being unloaded from the vehicle, with an average frequency for the 17 journeys of 32.8 ± 15.6% of horses showing these behaviors (min = 5.3%; max = 62.5%; median = 31.6%). The journeys showing horses losing balance at unloading were 14 (14/32, 43.75%) for a total of 78 horses (78/613, 12.7%) with an average frequency for the 14 journeys of 32.6 ± 18.2% of horses losing balance (min = 10.2%; max = 75%; median = 30.8%). Five horses (5/613, 0.8%) from five different journeys (5/32, 15.6%) fell during unloading. A total of 101 handled horses (101/613, 16.5%) from 17 journeys (17/32, 53.1%) showed sweating signs at unloading, with an average frequency for the 17 journeys of 18.9 ± 26% (min = 5.3%; max = 88.9%; median = 25.7%). Coughing was never noticed during unloading procedures. Four horses unloaded from three journeys (3/32, 9.4%) had a BCS = 1.

### 3.4. Descriptive Results of Horse Details and Their Welfare Status after Unloading

The transported horses were both draft-breed (387/606, 64%) and light-breed horses (219/606, 36%), mainly males (326/590, 55%), with an average age of 5 ± 7 years and an average BCS of 4 ± 1. Most of the horses transported to the slaughterhouse in Northern Italy were stabled in a long stall (257/293, 87.7%), where they remained tied with a long rope (about 1 m long) until they were headed to the stunning cage. The other horses were free to move (untied) in smaller single boxes of about 6.25 × 2.30 m. They had water and feed ad libitum and drank soon after unloading. The horses transported to the slaughterhouse in Southern Italy were tied with short ropes (about 60 cm long) in paddocks without shelters of about 15 × 15 m, containing 6 horses each. At the end of the unloading procedures and identification operations, the horses were released and maintained loose in the paddock, fed, and watered ad libitum. The bedding of the lairage facilities was always clean in both slaughterhouses. The average temperature in the stabling boxes/paddocks was 12.76 ± 7.48 °C, ranging from a minimum of 2 °C to a maximum of 30 °C (median = 11 °C). The average relative humidity was 39.87 ± 17.02%, ranging from 10% to 70% (median = 43%).

**Figure 2:**
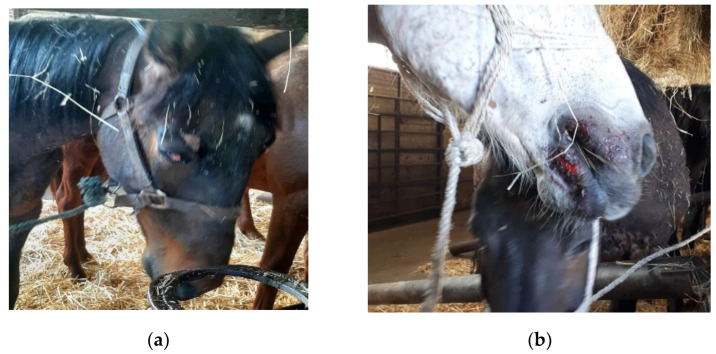
Two handled horses displaying injuries after being transported to the slaughterhouse: (**a**) example of an injury at the level of the orbital socket; (**b**) example of an injury at the level of lips.

All horses were alert and responsive or alert and calm, except for three lethargic horses, four horses that were depressed, two exhausted, and eight scared. The respiratory rate was 18.6 ± 4.1 bpm, ranging from a minimum of 10 bpm to a maximum of 44 bpm. Table 8 shows the prevalence of health parameters observed when animals were laired in the slaughterhouse. The most frequent health issues were injuries (151/613, 24.6%), nasal discharge (71/612, 11.6%), and lacrimal discharge (62/613, 10.1%). The injuries were located especially on horses’ heads or necks (103/613, 16.8%; Figure 2), on the orbital socket (51/613, 8.3%) (Figure 2a), or lips (31/613, 5.1%) (Figure 2b). Moreover, injuries on the tails (31/613, 5.1%), limbs (23/613, 3.8%), and trunk (16/613, 2.6%) were found. Among the horses transported to the Northern Italy slaughterhouse, more than half had a ruffled—but not injured—tail (164/314, 52.2%). Nasal discharges were bilateral in 48 horses (48/71, 67.6%) and purulent in two cases. Lameness was severe in two cases (score 4), four other animals were mildly lame (score 3), while the remaining were moderately or slightly lame (14/613; 3.4%). Three horses (3/613, 0.5%) were one-eye blind, and another three horses (3/613, 0.5%) showed abnormal feces. A stallion coming from Hungary, when stabled in the lairage stall, showed a repetitive movement of the front legs, like a Piaffe movement on site (Video S2). The horse did not show this abnormal behavior during unloading but was extremely aggressive towards conspecifics when stabled in the lairage stall and performed the behavior for the whole observation time. The stallion and the blind horses were therefore considered animals where fitness for transport was poorly assessed.

Horses with nasal and lacrimal discharge mainly came from France, with 50/71 (70.4%) and 46/62 (74.2%) horses, respectively. On the other hand, injured animals mainly came from Poland, with 120/151 (79.5%) horses traveling from Poland that had at least one injury. At unloading, horses reluctant to move/unload came mostly from Poland (101/136; 74.3%), as well as horses that were beaten with a stick or a rubber tube (40/50; 80%).

### 3.5. Associations between the Presence/Absence of Bilateral Nasal Discharge and Lacrimal Discharge

**Figure 3:**
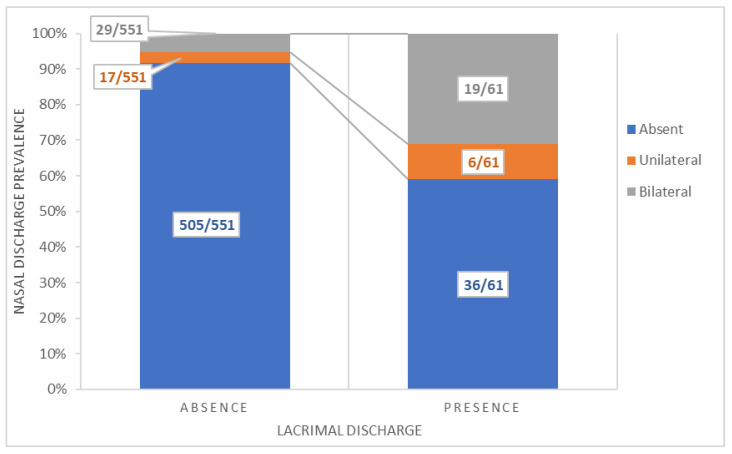
Association between the presence of lacrimal discharge and the severity of nasal discharge (absent, unilateral, or bilateral) in the 613 handled horses transported to two slaughterhouses in Italy.

Figure 3 shows the association found between the dummy outcome of lacrimal discharge and the severity (absence, unilateral, or bilateral) of nasal discharge. The presence and severity of nasal discharge were significantly associated with the presence of lacrimal discharge (*p* < 0.001). Horses with bilateral nasal discharge were nine times more likely to show lacrimal discharge after transport when compared with those without nasal discharge (OR = 9.19; 95% CI = 4.67–17.94; *p* < 0.001), and horses with unilateral nasal discharge were almost five times more likely to show lacrimal discharge (OR = 4.95; 95% CI = 1.70–12.75; *p* = 0.001). However, as shown in Figure 3, not all horses presenting lacrimal discharge also had nasal discharge, and 36 out of the 61 with lacrimal discharge (36/61; 59%) did not present comorbidity between nasal discharge and lacrimal discharge.

### 3.6. Risk Factors Associated with the Expression of Reluctance to Move/Unload Behavior at Unloading

The complete list of factors and the relative *p*-values for their associations in the univariable logistic regression models with the expression of reluctance to move/unload is reported in Table S6. Table 9 shows the OR, 95% CI, and *p*-values of the variables retained in the final multiple regression model for the horses being reluctant to move/unload at unloading (model *p*-value < 0.001, AIC = 485.98). The likelihood of observing horses expressing this behavior at unloading was sharply increased when handlers used the stick to beat the animals during unloading (*p* < 0.001). Horses were also more reluctant to move at unloading with increased unloading duration (*p* = 0.003) and when they had smaller BCS scores (*p* = 0.001). The season was also significantly associated with the expression of this behavior, with horses transported in autumn and winter being, respectively, six and two times more likely to display this behavior at unloading when compared with horses transported during spring (*p* < 0.001).

### 3.7. Risk Factors Associated with the Presence of Nasal Discharge

The complete list of factors and the relative *p*-values for their associations in the univariable logistic regression models with the presence of nasal discharge is reported in Table S7. Table 10 shows the OR, 95% CI, and *p*-values of the variables retained in the final multiple regression model for the presence of nasal discharge in the transported horses (model *p*-value < 0.001, AIC = 238.55). The likelihood of finding horses with nasal discharge at arrival at the slaughterhouse was higher when journeys and unloading phases were longer (*p* = 0.004 and *p* = 0.006, respectively), the transported horses had a lower BCS and were younger (*p* = 0.025 and *p* = 0.004, respectively), and when the arrival temperatures were lower (*p* = 0.001). Furthermore, the season was strongly associated with the prevalence of nasal discharge, with horses transported during spring being nineteen-times more likely to show this clinical sign when compared with winter (*p* < 0.001). Horses of the draft-breed type and transported tied were significantly more likely to have nasal discharge when compared with light-breed horses (*p* < 0.001) that were not tied during transport (*p* = 0.004). Vehicle change seems to be also a factor predisposing to the occurrence of nasal discharge (*p* = 0.024).

### 3.8. Risk Factors Associated with the Presence of Lacrimal Discharge

The complete list of factors and the relative *p*-values for their associations in the univariable logistic regression models with the presence of lacrimal discharge are reported in Table S8. Table 11 shows the OR, 95% CI, and *p*-values of the variables retained in the final multiple regression model for the presence of lacrimal discharge in the transported horses (model *p*-value < 0.001, AIC = 239.67). The likelihood of finding horses with lacrimal discharge was higher with increased arrival temperatures (*p* = 0.001) and was more than doubled when the horses were long-haired (*p* = 0.044), females (*p* = 0.010), and showed nasal discharge (*p* = 0.010).

### 3.9. Risk Factors Associated with the Presence of Cuts or Injuries on Horses’ Bodies

The complete list of factors and the relative *p*-values for their associations in the univariable logistic regression models with the presence of injuries or cuts on horses’ bodies are reported in Table S9. Table 12 shows the OR, 95% CI, and *p*-values of the variables retained in the final multiple regression model for the presence of cuts or injuries on the transported horses (model *p*-value < 0.001, AIC = 640.17). The season was strongly associated with the prevalence of injuries on horses’ bodies, with horses being transported in autumn, summer, and winter being, respectively, twenty-six, five, and three times more likely to show cuts, injuries, or bruises on their bodies when compared with spring (*p* < 0.001). Less driving experience was also significantly associated with an increased likelihood of finding injured horses at unloading (*p* = 0.016). Light-breed horses were also more likely to show injuries on the body (*p* = 0.019). The horse’s BCS was retained in the model as the AIC obtained including this variable in the multiple regression model was smaller than the AIC of the model without this factor (AIC = 641.26). The likelihood of finding injured horses seems therefore to be associated also with increased BCS (*p* = 0.069).

The complete list of factors and the relative *p*-values for their associations in the univariable logistic regression models with the presence of injuries or cuts on horses’ tails are reported in Table S10. Table 13 shows the OR, 95% CI, and *p*-values of the variables retained in the final multiple regression model for the presence of cuts or injuries on the transported horses’ tails (model *p*-value < 0.001, AIC = 184.22). The likelihood of observing horses with injured tails after unloading was higher when horses were transported for a longer period (*p* = 0.020) and when they were older draft-breed horses when compared with younger light-breed horses (*p* = 0.005). Furthermore, the likelihood of finding horses with injured tails was almost increased by nine times when handlers performed prohibited practices at unloading (*p* = 0.019).

The complete list of factors and the relative *p*-values for their associations in the univariable logistic regression models with the presence of injuries or cuts on horses’ heads and/or necks are reported in Table S11. Table 14 shows the OR, 95% CI, and *p*-values of the variables retained in the final multiple regression model for the presence of cuts or injuries on the transported horses’ heads and/or necks (model *p*-value < 0.001, AIC = 494.07). The likelihood of observing horses with injuries or cuts on their head and/or necks after unloading was higher with lower arrival temperatures (*p* < 0.001), and was almost ten times increased when horses experience a long stop during transport (*p* < 0.001).

## 4. Discussion

The present study described the routes, the journey conditions, and the welfare assessment of broken/handled horses traveling across Europe to be slaughtered in Northern and Southern Italy. The welfare status of the horses involved in the study was documented on arrival at the slaughterhouses, and after 30 min and 24 h after arrival, where possible, while the horses were kept in lairage pens/boxes, using a standardized protocol [22]. Our initial hypothesis was confirmed since associations between journey conditions and the welfare status of the horses on arrival were found. Journey duration, unloading duration, vehicle changes, long stops, handlers/drivers’ experience, temperature, season, and horse individual characteristics had a significant impact on horses’ welfare and health status [1,11,14]. Therefore, our study highlights the journey conditions that can represent a hazard to horses’ welfare and that should be duly considered for future revisions of any animal transport regulation. Our study also gives evidence that horse meat is commonly obtained not only from horses reared for meat purposes (i.e., draft horses) but often from light-breed horses, previously used for sports and then sent to slaughter (i.e., industry waste).

According to EC 1/2005 [16] adult and handled horses can travel by road for a maximum of 24 h, followed by 24 h of rest at a Control Post (CP), and must be watered and fed every 8 h. However, sport horses do not have to comply with these timing and journey breaks seem to be underregulated. During all the examined journeys, water and food were administered to the horses approximately every 8 h, in compliance with the regulation, but the journey breaks were very variable. In almost half of the cases, stops at the CPs were performed earlier (12 h ± 50 min) than the 24 h in transit and lasted about 12 h. A quarter of the examined journeys stopped at a CP only for two hours, during which the animals were unloaded, watered, fed, rested, and reloaded in a new vehicle. Finally, during two journeys, the horses rested for 9 h inside the vehicle, parked in a parking area, while they were also watered and fed. When the vehicle is stationary, adult horses cannot lie down, if they travel in a single bay, and are more likely to be exposed to heat stress [1] and poor welfare [12]. Overall, even if the exact timing for recovering after a long journey is still unclear, our findings suggest that increased attention should be devoted to the journey breaks in the enforcement of the regulation.

Stopping at the Control Post (CP) should be planned carefully and avoided when not needed [12]. Unloading/loading procedures and contact with horses coming from different farms may pose indeed additional welfare and epidemiological risks to horses’ health [4,12]. The fact that almost all the examined horses stopped at CP could be a factor in the prevalence of welfare issues found in our study. At the CP, a change in the vehicle can also add risk, as the animals are unloaded and reloaded onto a new vehicle, exposing them to new environmental conditions and to management-related vehicle biohazards (e.g., poor disinfection) [12,28]. However, it is important to highlight that stopping at CPs and changes of vehicles are not only dangerous for the horses but also for the horse handlers. Handling at loading/unloading represents a human-horse interaction that may pose potential risks for horse-related injuries to humans [29,30]. Overall, although the duration of the examined journeys complied with the EC 1/2005 [16], the journey planning is questionable and possibly exposed the horses to additional risks for their welfare. It is, therefore, necessary that regulations specify more precisely how the journey must be planned.

In this study, injuries were the most frequent welfare problem, followed by nasal and lacrimal discharge, while lameness, coughing, and abnormal feces were less frequently observed. The prevalence of discharge was in line with previous studies performed on horses transported to slaughterhouses in Italy [3] and Mexico [14]. The high prevalence of injured horses was in line with the median percentage of horses with acute injuries per shipment (24%) arriving at slaughterhouses in Italy found in a previous study [3]. These percentages of injured horses are, however, considerably higher than those reported in previous studies on handled [13,14,31] or unhandled horses [22]. This discrepancy may be partly explained by a different method to score injuries among studies, since we also counted shallow injuries, such as scars and bruises. However, the discrepancy could also be due to different methods to assess the fitness for transport, which has always been considered a major risk factor for the welfare of traveling horses [3,12]. In the present study, the researchers were not able to directly assess the fitness for transport, so our prevalence of injuries could be overestimated. After unloading, some horses were showing clinical problems (i.e., laminitis, neurological problems, blindness) which were judged preexisting since these severe and chronic health issues could not have been developed over 24 h, and some of the injuries could therefore have been also preexisting. It is worth highlighting that one-eye blindness is not considered among the unfitness requirements in the present regulations (Regulation EC 1/2005 [16]), and that requirements for unfitness are not unanimous among codes and regulations [20]. Overall, considering that the examined journeys were all approved by competent authorities, our findings suggest that the evaluation of the fitness for transport is still an area of concern and that many horses with pre-existing conditions are transported, leading to worse welfare on arrival [3]. A guideline on how to assess the fitness for transport, including a list of pathologies and health conditions to double-check and when to call the veterinarians, was published [12,32], but not enforced. Apart from the list of pathologies, there is a shortage of legislation covering fitness for transport on who is responsible to assess it. The responsibility of actors varies among the EU Member States, as some put equal responsibility on the keeper and the transporter, while others evaluate on a case-by-case basis [33]. In order to protect the welfare of animals during transport, European law should be enforced with a clear provision of responsibilities for the actors involved in the transport of live animals, and the assessment of fitness for transport should be entrusted to trained personnel.

Human-related factors have been considered among the main risk factors for poor welfare during horse transport. In our study, drivers with less experience were one of the human-related factors associated with horses’ injuries. Driver experience is of critical importance for the welfare and health of live animal transportation because more abrupt driving may predispose horses to have greater difficulty balancing during the journey [1,34], increasing the odds of finding non-ambulatory or markedly lame animals at unloading [34]. In the present study, less experienced drivers were associated with an increase in the prevalence of animals’ acute injuries, suggesting that lesser driving experience affected the animals’ ability to stay balanced during the journey. For safeguarding horses’ welfare during transport, better training of drivers carrying live animals is highly advisable and the laws should be enforced with mandatory and frequent refresher courses.

Improper handling of the horses during unloading/loading phases was another critical human-related factor that significantly increased the risk of animals being reluctant to move/unload [13] or showing injuries [11]. In our dataset, about 1 in 5 horses were reluctant to move at unloading, and the likelihood of having reluctant horses increased by more than two hundred times when handlers performed forbidden practices (such as using sticks to beat the animals), in agreement with previous studies [13]. This contrasts with the study by Zappaterra et al. [22], where the horses self-unloaded without showing any reluctance to move/unload. The possible explanation was that in the present study, the horses were all haltered and the improper use of negative reinforcement may have contributed to the increased performance of reluctance behavior [35]. Showing a stick or a rubber tube while unloading the horses has been proven to cause fear and therefore animals tend to stop and refuse to unload. Beating the animals with various aids is considered encouragement to get them moving, but was counterproductive [11,30]. Whipping or beating the horses increases stress levels, decreases their attention to the surrounding space and leads them to injure themselves more easily [11,30,36]. Moreover, improper handling and the frightened/reluctant to move/unload animals could have lengthened the duration of the unloading operations. Indeed, we found that prolonged unloading duration was associated with increased odds of finding horses being reluctant to move/unload. Prolonged unloading duration may indeed have increased the anxiety and fear in the horses still waiting inside the vehicle, resulting in a chain reaction in which the more animals were forced, the longer they took to unload, and the more the waiting animals experience anxiety and nervousness. Animals should be unloaded immediately after arrival, using handling based on animal learning theory or letting the animals self-unloaded quietly [12]. Moreover, better and more frequent training on animal welfare, care, and appropriate handling should be advisable for the operators working with horses at farms and slaughterhouses.

Transport conditions to slaughterhouses were also of critical importance for horses’ welfare. Horses transported for longer journeys, experiencing a vehicle change, and being tied during transport were most at risk of presenting health issues at unloading. The association between horses transported tied and an increased likelihood of showing nasal discharge at unloading confirmed previous results [11,12,14]. Restraint with a high head posture has been shown to predispose to inefficient airway clearance and increase the likelihood of developing respiratory diseases in horses [11,12,14]. In addition, the likelihood of finding horses with nasal discharge and cuts/injuries at unloading was increased by long journey duration. Longer journeys expose horses to a greater risk of coming into contact with pathogens since the conditions of high humidity and limited air circulation can increase the risk of contagion between animals. Furthermore, the longer the duration, the higher the risk of horses losing balance during transport [3]. The likelihood of developing injuries seems to be higher in the first 20 h of travel [7]. In the present study, journey durations were quite high, with a median duration value of 24 h, therefore confirming that horses being transported for about 20 h are particularly at risk of presenting health problems and injuries. However, in the present study, the health outcomes related to journey duration may also be related to other risk factors. Longer journey durations were collinear with Poland as the country of departure. Vehicles departing from Poland often stopped at the Control Posts. It is, therefore, possible that the duration of the transport, together with the performing of long stops, has predisposed the animals to have more health problems. Control Posts (referred also as designated border control posts) are places where official veterinarians check the health status of animals transported from different countries. At the Control Posts, animals are unloaded from vehicles, fed and watered, and they can rest before leaving again. However, this mixing of animals from different places, and with different clinical histories, can also increase the spreading of diseases. The risk that a pathological situation may arise is also increased by a lowering in the animals’ immune defenses due to transport and journey conditions [37]. These factors could therefore explain the association found between prolonged journey duration and the increased risk of nasal discharge in horses. Similarly, the horses experiencing a vehicle change during the journey toward the slaughterhouse were those that were more likely to have nasal discharge at unloading. Vehicle change likely predisposed the animals to greater stress and may also have increased the odds of horses coming into contact with pathogens and environmental conditions able to favor the onset of dysbiosis in the respiratory tract microbiome [38]. This study is the first to present data demonstrating that vehicle change is a quite common practice during the transport of horses to the slaughterhouse. The data obtained strongly suggest that this practice is risky as it is a hazard to horses’ health, exposing the animals to greater stress, caused by additional handling, unloading, loading and possible sudden changes in environmental conditions.

Arrival temperature and season were major factors affecting horses’ health and welfare. In particular, the animals transported during spring were more prone to show nasal discharge and health problems at unloading, in line with previous studies [7,39,40]. In European countries, spring is characterized by sudden temperature changes and higher relative humidity. The latter are risk factors increasing the likelihood of respiratory diseases spreading in several animal species, such as horses [41,42] and ruminants [39,43]. In addition to spring, lower temperatures on arrival were also found to be particularly associated with the presence of nasal discharge in the present study. This result is in line with previous studies, in which equid herpesviruses were identified at higher viral loads and in a greater number of horses during the winter season [41]. On the other hand, higher temperatures and travel during the summer were predisposing factors for lacrimal discharge. A dry climate increases dustiness, and dust may cause inflammatory responses in the nasopharyngeal tract, leading to conjunctivitis, or an excess of eye secretions to clear and lubricate the eyes. The animals considered in the present study showed watery and clear lacrimal secretions, suggesting that lacrimal secretions may be due to an attempt to remove dust from the eyes. Lacrimal discharge was also associated with the manifestation of nasal discharge. It is therefore possible that the presence of nasal discharge also predisposes to the development of lacrimal discharge, as found in other studies [14]. In general, it would be good to avoid as much as possible the sudden changes in temperature during the winter and spring periods, ensuring good environmental conditions inside the vehicle and keeping the animals in thermal comfort during the journey. To avoid temperature changes and dustiness inside the vehicle, it would also be advisable to maintain good ventilation during the summer, while avoiding strong air currents that could pose a risk to the horse’s health. This may be possible by using fully air-conditioned vehicles equipped with temperature monitoring and ventilation control systems, ensuring the maintenance of the effective temperature inside the vehicle within the thermoneutral zone [12]. Effective control of the environmental conditions inside the vehicle is therefore particularly important for maintaining a good level of welfare in the transported animals.

Among the factors considered in the present study, some horse individual characteristics, such as age, BCS and horse type, were significantly associated with certain health issues. For example, in the present study, younger horses had a higher likelihood of developing nasal discharge, and this is in line with what was found by Wood et al. [44]. Indeed, younger horses tend to be more predisposed to respiratory diseases than adults, which have a more mature immune system. On the other hand, adult draft-breed horses, with higher BCS, were significantly more predisposed to show cuts or injuries at arrival, in particular on the tail junction, the dock. It is worth noting that this type of problem was not identified in our previous study on unhandled draft-breed horses [22]. This could be because the unhandled horses were transported loose and in groups and therefore had more ability to balance during travel, while the handled horses considered in the present study were transported in individual bays, perpendicular to the direction of travel and often tied. The larger size of adult horses belonging to the draft-breed coupled with these journey conditions likely hindered their ability to balance during transport, and increased the likelihood of being injured. New solutions to limit these types of injuries are much needed. For example, the possibility of applying soft surfaces to the internal walls of the vehicle could be explored in dedicated studies, to cushion the horses during the journey and avoid injuries [8]. Moreover, as suggested by the EFSA opinion [12], larger lateral and front/back space in a single bay could improve horses’ capacity to maintain balance during transit, as well as being untied and traveling facing backward, thereby reducing the prevalence of possible injury. Further studies to test these solutions could also be of interest to other types of horses transported, such as leisure or sport horses.

Our study has limitations, and the results must be interpreted carefully. One of the main limitations is that we have no data on the horses’ fitness evaluation before departure. This hindered us in interpreting some welfare issues as pre-existing or related to transport. Furthermore, the protocol was only performed in two slaughterhouses. While this increases variability, some of the results may be strictly linked to the operating methods of that particular slaughterhouse and therefore cannot be generalized. Moreover, in the Southern Italy slaughterhouse, we did not obtain permission to video record the unloading phase, with the inability to double check the presence of some of the welfare consequences, such as ruffled tails. The study was performed for one year, but the distribution of the journeys was not regular. In addition, the evaluation of horses at unloading was performed at the group level and not on the individual horses. Finally, no diagnostic tests were carried out to confirm the presence of pathogens in the horses’ respiratory tracts. However, these limitations are in line with a field study where only feasible ABMs were recorded, minimizing what could be done during an official welfare inspection. Notwithstanding these limitations, this study has increased our knowledge of journey conditions and welfare issues related to long journeys toward slaughterhouses of handled horses in Europe. Our results, having highlighted the commonest hazardous journey conditions, provide evidence to implement the current regulations on the protection of welfare during the transport of handled horses.

## 5. Conclusions

This study describes the journeys, journey conditions, and welfare status of handled horses transported across Europe to slaughterhouses in Italy. No dead on arrival and only one severely injured horse were detected on arrival. Nevertheless, three horses were considered unfit for transport for orthopedic or behavioral problems. The prevalence of horses showing nasal discharge, lacrimal discharge, and/or injuries was in line with other investigations conducted on handled horses. Journey duration, unloading duration, vehicle changes, long stops, handlers/drivers’ skills, temperature, season, and horse individual characteristics were risk factors for the development of health issues, confirming that journey conditions can affect the welfare of handled horses traveling toward slaughterhouses. Appropriate journey planning, proper handling and driving, and horses traveling untied are very important factors to safeguard the welfare of handled horses during road transport. Our findings have provided evidence that can be useful for the improvement of current regulations on the protection of welfare during horse transport. However, further studies on the precise assessment of the fitness for transport before departure are needed.

## Figures and Tables

**Table 1:** List of information compared or retrieved from the official documentation.

Journey Log	TRACES	Horse Passport	CMR *
Time of departure	Departure country	Horse ID	Total load weight
Number of stops	Estimated journey duration **	Country of origin	
Length of the stops	Total load weight	Sex	
Animal rested, fed and watered		Age	
Vehicle change		Breed ***	
Time of arrival			

* = if present; ** = obtained from TRACES in case of missing information from the Journey Log; *** = used to categorize the horses as draft-breed or light-breed.

**Table 2:** Meaning and types of factors collected and included as factors in the subsequent statistical analyses.

Factors	Explanation	Types of Variables
**Journey-related factors**		
Duration from Journey log (h)	Journey duration from departure to arrival (from Journey Log)	Continuous
Classes of short stops (<12 h)	Number of short stops (<12 h) during the journey without horses unloading is categorized into two classes (between 0 and 2 short stops; more than 2 short stops)	Dichotomous
Long stops (>12 h)	Presence/absence of long stops (>12 h) during the journey.	Dichotomous
Stocking density (kg/m^2^)	How many kg of live weight was distributed per m^2^	Continuous
Tied	If the horses were tied in transit	Dichotomous
Season	Seasons in which horses traveled (i.e., Spring, Summer, Autumn, Winter)	Categorical
Fed during transport	If the horses were fed during transport	Dichotomous
Arrival temperature (°C)	External environmental temperature measured through a weather station	Continuous
Unloading duration (min)	From the lowering of the vehicle ramp until the last horse was unloaded measured with a stopwatch	Continuous
**Human-related factors**		
Driver’s experience (years)	Years of experience driving vehicles for the transport of horses	Continuous
The handler moves the animals properly	The handler moved the horse/s without endangering the safety of the animal/s or their own	Dichotomous
Use of the stick	The horse was beaten with a stick or a rubber tube at unloading	Dichotomous
**Vehicle-related factors**		
Ramp flooring	Cover material for the unloading ramp (i.e., Non-slip knurled metal with foot battens; Corrugated metal; Rubber mat)	Categorical
Vehicle floor bedding	Degree of coverage of the vehicle floor with bedding material (i.e., Partial, Complete)	Dichotomous
Vehicle bedding type	Bedding material for the vehicle floor (i.e., Straw, Shavings, Straw and shavings, Sand and shavings)	Categorical
Type of drinkers	Structure and characteristics of the drinkers (i.e., Portable, Fixed bowls)	Dichotomous
**Horse-related factors**		
Horse’s Age	Age of the individual horse	Continuous
Horse’s Type	Categorization of morphology (i.e., light-breed horse, draft-breed horse)	Dichotomous
Horse’s age*Horse’s type	Interaction between horse’s age (continuous) and horse’s type (dichotomous)	Continuous inside each class
Horse’s Body Condition Score (BCS)	Number of horses per vehicle that present 0–1 or 5 score BCS	Continuous
Coat	Presence of winter (long) or summer (short) hair on the animal	Dichotomous

**Table 3:** Meaning and types of outcome variables concerning horse’s health and welfare-related parameters recorded at unloading or after arrival at the slaughterhouses.

Outcome	Explanation	Type
Reluctance to move/unload	The horse showed unwillingness to move forward or move from its position during unloading per journey	Dichotomous
Injuries	Presence/absence of injuries (cuts and/or bruises on the animal’s body)	Dichotomous
Injured tail	Presence/absence of injuries on the tail	Dichotomous
Injured head/neck	Presence/absence of injuries on the head and/or neck	Dichotomous
Nasal discharge	Presence/absence of loss of material from the nose	Dichotomous
Lacrimal discharge	Presence/absence of loss of material from the eyes	Dichotomous

**Table 4:** Routes, long and short stops (duration and location), total journey duration, transit stage duration, arrival time and temperature (T), and change of vehicle for each assessed journey.

Journey ID	Vehicle ID	Route	Long Stops	Short Stops	Journey Duration *	Hours in Transit	Arrival Time	Arrival T (°C)	Vehicle Change	Notes
1	1	POL-ITA	1 (12 h—CP): HRV	1st (1 h): CZE; 2nd (1 h): ITA	39 h	25 h 30′	9:30	36	No	Watering during short stops. Watering and feeding at CP
2	1	POL-ITA	1 (12 h—CP): HRV	1st (1 h): CZE; 2nd (1 h): ITA	40 h	26 h	11:00	35	No	Watering during short stops. Watering and feeding at CP
3	1	POL-ITA	1 (12 h—CP): HRV	1st (1 h): CZE; 2nd (1 h): ITA	39 h	25 h	12:00	32	No	Watering during short stops. Watering and feeding at CP
4	1	POL-ITA	1 (12 h—CP): HRV	1st (1 h): CZE; 2nd (1 h): ITA	40 h 40′	26 h 40′	9:30	25	No	Watering during short stops. Watering and feeding at CP
5	1	POL-ITA	1 (12 h—CP): HRV	1st (1 h): CZE; 2nd (1 h): ITA	40 h	26 h	10:00	31	No	Watering during short stops. Watering and feeding at CP
6	1	POL-ITA	1 (12 h—CP): HRV	1st (1 h): CZE; 2nd (1 h): ITA	41 h	27 h	10:30	28	No	Watering during short stops. Watering and feeding at CP
7	1	POL-ITA	1 (12 h—CP): HRV	1st (1 h): CZE; 2nd (1 h): ITA	40 h 30′	26 h 30′	9:45	24	No	Watering during short stops. Watering and feeding at CP
8	1	POL-ITA	1 (12 h—CP): HRV	1st (1 h): CZE; 2nd (1 h): ITA	41 h 45′	27 h 45′	10:15	24	No	Watering during short stops. Watering and feeding at CP
9	1	POL-ITA	1 (12 h—CP): HRV	1st (1 h): CZE; 2nd (1 h): ITA	41 h 15′	27 h 15′	09:30	27	No	Watering during short stops. Watering and feeding at CP
10	1	POL-ITA	1 (12 h—CP): HRV	1st (1 h): CZE; 2nd (1 h): ITA	42 h 5′	28 h 5′	10:15	24	No	Watering during short stops. Watering and feeding at CP
11	1	POL-ITA	1 (12 h—CP): HRV	1st (1 h): CZE; 2nd (1 h): ITA	41 h 30′	27 h 30′	10:00	22	No	Watering during short stops. Watering and feeding at CP
12	1	POL-ITA	1 (12 h—CP): HRV	1st (1 h): CZE; 2nd (1 h): ITA	42 h 10′	28 h 10′	9:40	21	No	Watering during short stops. Watering and feeding at CP
13	1	POL-ITA	1 (12 h—CP): HRV	1st (1 h): CZE; 2nd (1 h): ITA	41 h 45′	27 h 45′	9:45	17	No	Watering during short stops. Watering and feeding at CP
14	1	POL-ITA	1 (12 h—CP): HRV	1st (1 h): CZE; 2nd (1 h): ITA	42 h 45′	28 h 45′	10:30	21	No	Watering during short stops. Watering and feeding at CP
15	1	POL-ITA	1 (12 h—CP): HRV	1st (1 h): CZE; 2nd (1 h): ITA	40 h 30′	26 h 30′	9:40	19	No	Watering during short stops. Watering and feeding at CP
16	2	FRA-ITA	0	1st (45′): FRA; 2nd (2 h—CP): FRA; 3rd (1 h 15′): ITA	19 h 30′	15 h 30′	13:00	17	Yes (2nd stop)	Watering and feeding at CP
17	2	FRA-ITA	0	1st (30′): FRA; 2nd (2 h—CP): FRA; 3rd (1 h 45′): ITA	19 h 30′	15 h 15′	13:00	13	Yes (2nd stop)	Watering and feeding at CP
18	3	POL-ITA	0	2: 1st (1 h): POL; 2nd (1 h): HUN	20 h 40′	18 h 40′	14.40	8	No	Waiting for 30′ before unloading the horses
19	2	FRA-ITA	0	1st (45′): FRA; 2nd (2 h—CP): FRA; 3rd (45′): FRA; 4th (45′): ITA	19 h 45′	15 h 30‘	14:00	13	Yes (2nd stop)	Watering and feeding at CP
20	2	FRA-ITA	0	1 (45′): ITA	11 h	10 h 15′	17:00	8	No	None
21	4	FRA-ITA	0	1st (30′): FRA; 2nd (9 h): FRA; 3rd (45′): Frau	25 h 30′	15 h 15′	15:20	11	No	Watering and feeding during 2nd stop (parking area)
22	2	FRA-ITA	0	1st (30′): FRA; 2nd (2 h—CP): FRA; 3rd (1 h 10′): ITA	17 h 50′	14 h 10′	11:50	13	Yes (2nd stop)	Watering and feeding at CP
23	5	SLO-ITA	0	No information °	Missing	Missing	15:00	15	No	No Journey Log (<8 h)
24	2	FRA-ITA	0	1st (45′): FRA; 2nd (2 h—CP): FRA; 3rd (1 h 20′): ITA	19 h 30′	15 h 25′	13:00	19	Yes (2nd stop)	Watering and feeding at CP
25	6	POL-ITA	0	1st (1 h): HUN; 2nd (1 h): HUN	23 h	21 h	15:00	19	No	None
26	2	FRA-ITA	0	1 (45′): ITA	9 h 30′	8 h 45′	17:00	14	No	None
27	2	FRA-ITA	0	1st (45′): FRA; 2nd (2 h—CP): FRA; 3rd (1 h 30′): ITA; 4th (30′): ITA	20 h 30′	15 h 45′	13:30	13	Yes (2nd stop)	Watering and feeding at CP
28	2	FRA-ITA	0	1st (45′): FRA; 2nd (2 h—CP): FRA; 3rd (45′): FRA; 4th (45′): ITA	21 h 05′	16 h 50′	13:35	18	Yes (2nd stop)	Watering and feeding at CP
29	7	HUN-ITA	0	No information °	9 h 45′	Missing	16:15	17	No	None
30	4	FRA-ITA	0	1st (30′): FRA; 2nd (9 h): FRA; 3rd (45′): Frau	26 h 5′	15 h 50′	18:15	17	No	Watering and feeding during 2nd stop (parking area)
31	2	FRA-ITA	0	1st (45′): FRA; 2nd (2 h—CP): FRA; 3rd (45′): ITA	19 h 50′	16 h 20′	13:20	22	Yes (2nd stop)	Watering and feeding at CP
32	5	SLO-ITA	0	No information °	Missing	Missing	14:55	22	No	No Journey Log (<8 h). Waiting for 20′ before unloading the horses

Legend: * = based on Journey Log; POL = Poland; ITA = Italy; HRV = Croatia; CZE = Czech Republic; FRA = France; SLO = Slovenia; HUN = Hungary; CP = Control Post; ° = unclear and non-objective data.

**Table 5:** Descriptive statistics for the journey durations and load weight observed from the documentation of the 32 journeys of handled horses towards the two Italian slaughterhouses.

Journey Duration and Load Weights	Mean ± S.D.	Min	Median	Max	Missing Data
Duration from TRACES (h)	26.5 ± 14	5	24	42	0
Duration from Journey Log (h)	29.6 ± 12	8	32.5	42.5	2
Hours in transit (h)	21 ± 6.5	8	23	28.5	2
Short stops (n)	2.4 ± 0.8	1	2	4	3
Short stops duration (min)	77 ± 82	30	60	540	3
Load weight from CMR (kg)	10,762 ± 2409	4950	10,957	13,800	3
Load weight from TRACES (kg)	9833 ± 2048	5120	9880	13,700	12

**Table 6:** Descriptive statistics for the variables related to driver’s information and journey conditions.

Variables	Mean ± S.D.	Min	Median	Max
Arrival temperature (°C)	20.2 ± 7.2	8	19	36
**Driver’s information**				
Driver’s age (years)	54 ± 4	46	55	58
Driver’s experience (years)	20 ± 6	15	17	31
**Journey conditions**				
Total vehicle dimension (m^2^)	34.10 ± 3.66	16.66	34	35.90
Space allowance (m^2^/animal)	1.74 ± 0.09	1.63	1.71	1.99
Stocking density (kg/m^2^)	323.88 ± 49.84	203.92	339.83	384.40
Number of forced ventilators	8.79 ± 6.78	2	6	20
Drinkers (n)	18.39 ± 7.26	0 *	20	22
Horses per vehicle (n)	19.61 ± 2.49	10	19	22

* = 0 is for the vehicles equipped with portable drinkers.

**Table 7:** Frequency table of journeys (n = 32) and handled horses (n = 613) for the categorical variables related to the vehicle and journey characteristics.

Variables	Journeys Count (%)	Horses Count (%)
Departure country:		
France	12/32 (37.5%)	223/613 (36.4%)
Poland	17/32 (53.1%)	356/613 (58%)
Other Eastern European	3/32 (9.4%)	34/613 (5.6%)
Presence of cameras:		
No	19/32 (59.4%)	380/613 (62%)
Yes	13/32 (40.6%)	233/613 (38%)
Ramp flooring:		
Non-slip knurled metal with foot battens Corrugated metal or rubber mat	16/32 (50%)	274/613 (44.7%)
	16/32 (50%)	339/613 (55.3%)
Ramp side gates:		
No	1/32 (3.1%)	19/613 (3%)
Yes	31/32 (96.9%)	605/613 (97%)
Ramp covering:		
None	16/32 (50%)	274/613 (44.7%)
Partial	16/32 (50%)	339/613 (55.3%)
Vehicle bedding type:		
Straw	17/31 (58.8%)	358/614 (58.3%)
Shavings	14/31 (45.2%)	256/614 (45.7%)
Type of drinkers:		
Fixed bowls	27/32 (84.4%)	544/613 (87.2%)
Portable	5/32 (15.6%)	80/613 (12.8%)
Water tank:		
Partially Empty	24/27 (88.9%)	487/529 (92.1%)
Empty	3/27 (11.1%)	42/529 (7.9%)
Lighting system for animals’ orientation:		
No	17/32 (53.1%)	349/613 (56.9%)
Yes	15/32 (46.9%)	264/613 (43.1%)
Tied:		
No	12/32 (37.5%)	223/613 (36.4%)
Yes	20/32 (62.5%)	390/613 (63.6%)
Fed during transport:		
Yes	25/32 (78.1%)	509/613 (83%)
No	7/32 (21.9%)	104/613 (17%)
Long stops (>12 h):		
No	17/32 (53%)	293/613 (47.8%)
Yes	15/32 (47%)	320/613 (52.2%)
Number of short stops classes:		
Between 0 and 2	21/30 (70%)	419/589 (71.1%)
More than 2	9/30 (30%)	170/589 (28.9%)
Vehicle change:		
No	23/32 (71.9%)	442/613 (72.1%)
Yes	9/32 (28.1%)	171/613 (27.9%)
The handler performs prohibited practices:		
Yes	17/32 (53.1%)	356/613 (58.1%)
No	15/32 (46.9%)	257/613 (41.9%)
Season:		
Spring	9/32 (28%)	151/613 (24.6%)
Summer	10/32 (31%)	215/613 (35%)
Autumn	5/32 (16%)	105/613 (17.1%)
Winter	8/32 (25%)	146/613 (23.2%)

**Table 8:** Prevalence of health parameters for the handled horses (n = 613) and 32 journeys towards slaughterhouses in Italy.

Health Parameters	Journeys Count (%)	Horses Count (%)
Injuries/cuts	28/32 (87.5%)	151/613 (24.6%)
Nasal discharge	16/32 (50%)	71/612 (11.6%)
Lacrimal discharge	14/32 (43.7%)	62/613 (10.1%)
Lameness	10/32 (31.3%)	20/613 (2.3%)
Coughing	3/32 (9.4%)	6/613 (1%)
Abnormal feces	2/32 (6.3%)	3/613 (0.5%)
One-eye blind	3/32 (9.4%)	3/613 (0.5%)

**Table 9:** Reluctance to move/unload—final multiple regression model for the dummy dependent variable of presence/absence of handled horses showing reluctant to move/unload behavior at arrival at the slaughterhouse. Data are presented as estimates and standard errors (S.E.) of the effect, odds ratios (OR), confidence interval (95% CI), and *p*-value. Bold *p*-values refer to the statistical significance of the predictive variable in the model; the significance of a category against the reference is reported in regular font.

Factors	Estimate ± S.E.	OR (95% CI)	*p*-Value
Unloading duration	0.285 ± 0.097	1.33 (1.10–1.61)	**0.003**
Season:			**<0.001**
Spring	Ref		
Summer	0.500 ± 0.437	1.65 (0.71–3.99)	n.s.
Autumn	1.787 ± 0.459	5.97 (2.49–15.12)	<0.001
Winter	0.797 ± 0.375	2.22 (1.08–4.75)	0.034
Beaten with a stick:			**<0.001**
No	Ref		
Yes	5.428 ± 1.037	227.66 (46.30–4139.89)	<0.001
Horse’s BCS	−0.308 ± 0.096	0.73 (0.61–0.88)	**0.001**

Ref: reference class; n.s.: not significant.

**Table 10:** Nasal discharge—final multiple regression model for the dummy dependent variable of presence/absence of nasal discharge in handled horses at arrival at the slaughterhouse. Data are presented as estimates and standard errors (S.E.) of the effect, odds ratio (OR), confidence interval (95% CI), and *p*-value. Bold *p*-values refer to the statistical significance of the predictive variable in the model; the significance of a category against the reference is reported in regular font.

Factors	Estimate ± S.E.	OR (95% CI)	*p*-Value
Arrival temperature	−0.330 ± 0.103	0.72 (0.58–0.87)	**0.001**
Duration from Journey Log	0.128 ± 0.063	1.14 (1.00–1.29)	**0.004**
Horse’s age	−0.045 ± 0.023	0.96 (0.91–1.00)	**0.004**
Horse’s BCS	−0.252 ± 0.163	0.77 (0.56–1.00)	**0.025**
Unloading duration	0.199 ± 0.105	1.22 (1.00–1.50)	**0.006**
Tied:			**0.015**
No	Ref		
Yes	1.334 ± 0.794	3.79 (0.11–18.54)	0.017
Vehicle change:			**0.024**
No	Ref		
Yes	0.810 ± 0.524	2.25 (0.82–6.50)	0.049
Season:			**<0.001**
Winter	Ref		
Spring	2.950 ± 0.568	19.11 (6.54–61.48)	<0.001
Autumn	-	-	n.s.
Summer	-	-	n.s.
Horse’s type:			**<0.001**
Light-breed	Ref		
Draft-breed	2.026 ± 0.716	7.58 (1.94–3.26)	0.004

Ref: reference class; n.s.: not significant.

**Table 11:** Lacrimal discharge—final multiple regression model for the dummy dependent variable of presence/absence of lacrimal discharge in handled horses at arrival at the slaughterhouse. Data are presented as estimates and standard error (S.E.) of the effect, odds ratio (OR), confidence interval (95% CI), and *p*-value. Bold *p*-values refer to the statistical significance of the predictive variable in the model; the significance of a category against the reference is reported in regular font.

Factors	Estimate ± S.E.	OR (95% CI)	*p*-Value
Arrival temperature	0.186 ± 0.059	1.20 (1.07–1.36)	**0.001**
Horse’s sex:			**0.010**
Male	Ref		
Female	0.898 ± 0.365	2.45 (1.22–5.18)	0.014
Coat:			**0.044**
Short	Ref		
Long	0.699 ± 0.348	2.01 (1.01–3.98)	0.045
Nasal discharge:			**0.010**
No	Ref		
Yes	0.917 ± 0.354	2.50 (1.24–5.00)	0.010

Ref: reference class.

**Table 12:** Injured horses—final multiple regression model for the dummy dependent variable of presence/absence of injured horses at arrival at the slaughterhouse. Data are presented as estimates and standard errors (S.E.) of the effect, odds ratio (OR), confidence interval (95% CI), and *p*-value. Bold *p*-values refer to the statistical significance or trend towards the significance of the predictive variable in the model; the significance of a category against the reference is reported in regular font.

Factor	Estimate ± S.E.	OR (95% CI)	*p*-Value
Driver’s experience	−0.262 ± 0.108	0.77 (0.62–0.95)	**0.016**
Horse’s BCS	0.175 ± 0.096	1.19 (0.99–1.44)	**0.069**
Horse’s type:			**0.019**
Draft-breed	Ref		
Light-breed	1.343 ±0.607	3.83 (1.25–13.90)	0.027
Season:			**<0.001**
Spring	Ref		
Summer	1.619 ± 0.608	5.04 (1.69–18.82)	0.008
Autumn	3.270 ± 0.659	26.30 (8.03–109.02)	<0.001
Winter	1.266 ± 0.509	3.55 (1.34–9.98)	0.013

Ref: reference class.

**Table 13:** Injured tails—final multiple regression model for the dummy dependent variable of presence/absence of injured tails in horses at arrival at the slaughterhouse. Data are presented as estimates and standard errors (S.E.) of the effect, odds ratio (OR), confidence interval (95% CI), and *p*-value. Bold *p*-values refer to the statistical significance of the predictive variable in the model; the significance of a category against the reference is reported in regular font.

Factor	Estimate ± S.E.	OR (95% CI)	*p*-Value
Duration from Journey Log	0.134 ± 0.066	1.14 (1.01–1.31)	**0.020**
The handler performs prohibited practices:			**0.019**
No	Ref		
Yes	2.170 ± 0.964	8.76 (1.25–61.00)	0.024
Horse’s age x Horse’s type:			**0.005**
Younger light-breed horses	Ref		
Older draft-breed horses	0.199 ±0.087	1.22 (1.05–1.48)	0.022

Ref: reference class.

**Table 14:** Injured head/neck—final multiple regression model for the dummy dependent variable of presence/absence of injuries on the head and/or neck of horses at arrival at the slaughterhouse. Data are presented as estimates and standard errors (S.E.) of the effect, odds ratio (OR), confidence interval (95% CI), and *p*-value. Bold *p*-values refer to the statistical significance or trend towards the significance of the predictive variable in the model; the significance of a category against the reference is reported in regular font.

Factor	Estimate ± S.E.	OR (95% CI)	*p*-Value
Arrival temperature	−0.132 ± 0.038	0.88 (0.81–0.94)	**<0.001**
Long stops (>12 h):			**<0.001**
No	Ref		
Yes	2.294 ± 0.308	9.91 (4.77–21.30)	<0.001

Ref: reference class.

## Data Availability

The data presented in this study are available on request from the corresponding author.

## Supplementary Materials

The following supporting information can be downloaded at: https://www.mdpi.com/article/10.3390/ani12223122/s1, Table S1: Checklist used during unloading to evaluate horses’ welfare at arrival (reported in Zappaterra et al. [22], modified from Messori et al. [13]); Table S2: Checklist used within 30 min of the unloading of the last animal to evaluate the individual horses’ welfare status at the lairage facilities (reported in Zappaterra et al. [22], modified from Messori et al. [13]); Table S3: Checklist used after unloading to obtain information about the driver, vehicle and documentation (reported in Zappaterra et al. [22], modified from Messori et al. [13]); Table S4: Checklist used 24 h after unloading to evaluate the individual horse welfare status at the lairage facilities (reported in Zappaterra et al. [22]); Table S5: Meaning and types of variables collected and statistically analyzed in the present study (modified from Zappaterra et al. [22]); Table S6: Associations between the predictive variables and the presence/absence of reluctance to move/unload behavior at unloading in the 613 handled horses transported to two slaughterhouses in Italy. Associations are presented with Wald Test *p*-values; Table S7: Associations between the predictive variables and the presence/absence of nasal discharge in the 613 handled horses transported to two slaughterhouses in Italy. Associations are presented with Wald Test *p*-values; Table S8: Associations between the predictive variables and the presence/absence of lacrimal discharge in the 613 handled horses transported to two slaughterhouses in Italy. Associations are presented with Wald Test *p*-values; Table S9: Associations between the predictive variables and the presence/absence of injuries and cuts in the 613 handled horses transported to two slaughterhouses in Italy. Associations are presented with Wald Test *p*-values; Table S10: Associations between the predictive variables and the presence/absence of injured tails in the 613 handled horses transported to two slaughterhouses in Italy. Associations are presented with Wald Test *p*-values; Table S11: Associations between the predictive variables and the presence/absence of injured head/neck in the 613 handled horses transported to two slaughterhouses in Italy. Associations are presented with Wald Test *p*-values; Video S1: Unloading of a horse coming from Slovenia and deemed unfit for transport; Video S2: The repetitive pattern of movements observed in a stallion transported from Slovenia.
