# Supplementary material for: Journeys, Journey Conditions, and Welfare Assessment of Broken (Handled) Horses on Arrival at Italian Slaughterhouses
Source: Animals (Basel). 2022 Nov 12;12(22):3122. doi: 10.3390/ani12223122 (PMC9686993; doi:10.3390/ani12223122)
Supplement: Supplementary file 1 [file animals-12-03122-s001.zip › Tables S1-S11.pdf]

Table S1. Checklist used during unloading to evaluate horses' welfare at arrival (reported in Zappaterra et al. [22], modified from Messori et al. [13]).

	Variable	Observations
UNLOADING PHASE: one copy for each journey	Arrival temperature and humidity	_ _ _ _ _ _
	Date and time of arrival	_ _ _ _ _ _
	Duration of unloading	_ _ _ _ _ _ _ _ _ minutes
	Total animals transported	_ _ _ _ _ _ _ N horses
	Dead on arrival (DOA)	_ _ _ _ _ _ _N horses
	Non-ambulatory*	_ _ _ _ _ _ _N horses
	Reluctant to move/unload*	_ _ _ _ _ _ _ __N horses
	Lame horses*	_ _ _ _ _ _ _N horses
	Slipping/Losing balance	_ _ _ _ _ _ _ _ N horses
	Falling	_ _ _ _ _ _ _ _ N horses
	Galloping or jumping	_ _ _ _ _ _ _ _ N horses
	Body Condition Score	Score 0-1:_ _ _ N horses<br>Score 5:_ _ _ N horses
	Sweating	_ _ _ _ _ _ _ _ N horses
	Injuries	_ _ _ _ _ _ _ _ N horses
	Exhaustion	_ _ _ _ _ _ _ _ N horses
	Coughing (more than one event)	□ Yes □ No
	The handler moves the animals in an arousing manner, inducing fear	□ Yes □ No
	The handler moves the animals in a noisy manner, inducing fear	□ Yes □ No
	The handler hits the animals without reason	□ Yes □ No
	The handler moves the animals properly**	□ Yes □ No
	The handler performs prohibited practices**	□ Yes □ No

* mutually exclusive; ** mutually exclusive; "Non-ambulatory" = unable to independently assume and maintain a standing position; "Reluctant to move/unload" = unwillingness to move forward or move from its position; "Lame horses" = obvious attempt to take the weight off one or more limbs; "Losing balance" = slipping without the body hitting the ground; "Injuries" = damage to tissues, at the skin level or deeper.

Table S2. Checklist used within 30 minutes of the unloading of the last animal to evaluate the individual horses' welfare status at the lairage facilities (reported in Zappaterra et al. [22], modified from Messori et al. [13]).

	**Date________ Hour________ Venue________ Vehicle ID________________ Horse ID________ Duration of travel________ Country of origin_________________**	
**INDIVIDUAL HORSE AFTER UNLOADING (to be filled for each horse within 30 minutes after unloading)**	Sex	□ Male □ Female
	Halter	□ Yes □ No
	Age	_ _ _ _ _ _ _ _
	Horse type	□ Draft-breed □ Light-breed
	Body Condition Score	_ _ _ _ _ _ _ _
	Estimated weight	_ _ _ _ _ _ _ _
	Demeanor	□ Alert, responsive<br>□ Alert, quite<br>□ Lethargic<br>□ Depressed<br>□ Exhausted<br>□ Terrified
	Heart rate	_ _ _ _ _ _ _ _

Respiratory rate	_ _ _ _ _ _ _ _
Coat	□ Long □ Short
Sweating	□ Yes □ No
Feces	□ Normal<br>□ Not formed<br>□ Diarrhoea
Clinical signs of colic	□ Yes □ No
Tail	□ Normal<br>□ Ruffled<br>□ Visible skin<br>□ Split, with damaged skin
Nasal discharge	□ Bilateral<br>□ Unilateral<br>□ None
Type of nasal discharge	□ Watery<br>□ Purulent<br>□ Bloody mucus<br>□ Smelly
Other discharges	□ Eyes<br>□ Mouth<br>□ Rectum<br>□ Vulva or penis<br>□ None
Cough	□ Yes □ No
Cuts or injuries	□ Yes □ No<br>How many_ _ _ _ _ _<br>Which_ _ _ _ _ _
Lameness score	□ 0 □ 1 □ 2 □ 3 □ 4 □ 5
Other clinical signs	□ Yes_ _ _ _ _ _ _ _ □ No
Notes	_ _ _ _ _ _ _ _

Table S3. Checklist used **after unloading** to obtain information about the driver, vehicle and documentation (reported in Zappaterra et al., [22], modified from Messori et al. [13]).

	Variable	Observations
DRIVER	Age	_ _ _ _ _ _ _ _
	Years of experience	_ _ _ _ _ _ _ _
	Certificate of competence	□ Yes □ No
VEHICLE AND RAMP	Date________ Hour________ Venue________	External temperature________ Humidity________
	Vehicle ID________	Vehicle origin________
	Vehicle covering	□ Yes □ No
	Forced ventilation	□ Yes (number)_ _ _ _ □ No
	Cameras	□ Yes (number)_ _ _ _ □ No
	Horses in group:	□ Yes Group number_ _ _ _<br>□ No
	If tied:	Rope length (cm) _ _ _ _<br>□ Appropriate<br>□ Inadequate
	Deck height	(cm) _ _ _ _ _ _ □ Appropriate □ Inadequate
	Ramp height	(cm) _ _ _ _ _ _ □ Appropriate □ Inadequate
	Direction of travel	□ 90° (head left, right), □ 45° (head left, right)<br>□ Forward, □ Backwards
	Adjacent stalls*	□ Yes □ No
	Ramp flooring type	_ _ _ _ _ _ _ _
	Ramp flooring conditions	□ Appropriate □ Inadequate

Ramp covering	□ Bedding completely covering<br>□ Bedding partially covering □ None
Ramp lateral protections	□ Yes, without openings or sharp edges<br>□ Yes, with openings or sharp edges □ No
Slots/Steps (> 10 cm between vehicle/ramp/floor/doors)	□ Yes □ No
Ramp slope	□ Compliant □ Not compliant
Blockage zones (holes or physical obstacles) on the ramp	□ Yes □ No
Lighting for animal orientation	□ Yes □ No
Lighting for animal movement during unloading	□ Yes □ No
Sharp edges	□ Yes □ No
Number of partitions	_ _ _ _ _ _ _ _
Dimensions of bays/compartments	Width_ _ _ _ _ _ _ Length_ _ _ _ _ _ _ _
Dimensions of the vehicle (m^2)	_ _ _ _ _ _ _ _
Vehicle floor covering	□ Bedding completely covering<br>□ Bedding partially covering □ None
Drinkers	Total number_ _ _ _ Working number_ _ _ _ _
Drinker type	_ _ _ _ _ _ _ _
Water tank	□ Full □ Partially Empty □ Empty
Temperature monitoring and control systems	□ Yes, working_ _ _ _ _ _ _ _(°C)<br>□ Yes, not working □ None
Signs of diarrhea in the vehicle	□ Yes □ No
Documents/information checklist	□ Passport, □ CMR, □ TRACES, □ Journey Log, □ Weight of the load

* Presence of horses in adjacent bays that cannot travel close together safely (mare/stallion or stallion/stallion).

Table S4. Checklist used 24 h after unloading to evaluate the individual horse welfare status at the lairage facilities (reported in Zappaterra et al. [22]).

	**Date________ Hour________ Venue________ Vehicle ID________ Horse ID________**<br>**Duration of travel________ Country of origin________**	
**INDIVIDUAL HORSE (24 h after unloading)**	Paddock/stabling box temperature	_ _ _ _ _ _ _ _
	Paddock/Stabling humidity	_ _ _ _ _ _ _ _
	Paddock/stabling exposure	□ Sun □ Shadow
	Number of horses per paddock/box	_ _ _ _ _ _ _ _ horses
	Bedding cleaning	□ Clean<br>□ Partially clean<br>□ Dirty
	Freedom of movement	□ Yes □ No
	Sex	□ Male □ Female
	Age	_ _ _ _ _ _ _ _
	Horse type	□ Draft-breed<br>□ Light-breed
	Declared level of animal handling	□ Broken □ Unbroken
	Demeanor	□ Alert, responsive<br>□ Alert, quite<br>□ Lethargic<br>□ Depressed<br>□ Exhausted<br>□ Scared
	Heart rate	_ _ _ _ _ _ _ _
	Respiratory rate	_ _ _ _ _ _ _ _

Feces	□ Normal<br>□ Not formed<br>□ Diarrhea
Clinical signs of colic	□ Yes □ No
Nasal discharge	□ Bilateral<br>□ Unilateral<br>□ None
Type of nasal discharge	□ Watery<br>□ Purulent<br>□ Bloody mucus<br>□ Smelly
Other discharges	□ Eyes □ Mouth<br>□ Rectum □ Vulva or penis □ None
Cough	□ Yes □ No
Cuts or injuries (including tail)	□ Yes □ No<br>How many_ _ _ _ _ _<br>Which_ _ _ _ _ _
Lameness score	□ 0 □ 1 □ 2 □ 3 □ 4 □ 5
Other clinical signs	□ Yes_ _ _ _ _ _ _ _ □ No
Broken/unbroken test (BUT)	Approaching and haltering □ 0 □ 1 □ 2<br>Handling □ 0 □ 1 □ 2
Notes	_ _ _ _ _ _ _ _

Table S5. Meaning and types of variables collected and statistically analysed in the present study (Table 5 of Zappaterra et al. [22]).

Variables	Explanation	Types of variables
**Journey-related variables**		
Country of origin	Country of birth of the horse (i.e., France; Poland; Slovenia, Czech Republic; Croatia; UK; Ireland; Holland; Germany)	Categorical (3 classes: France; Poland; Others)
Departure country	Country of departure of each journey (i.e., France; Poland; Slovenia; Hungary)	Categorical (3 classes: France; Poland; Other Eastern countries)
Duration from TRACES (h)	Journey duration from departure to arrival (from TRACES)	Continuous
Duration from Journey log (h)	Journey duration from departure to arrival (from Journey Log)	Continuous
Hours in transit (h)	Number of hours spent by the horses on the vehicle in motion (stops excluded)	Continuous
Number of short stops (<12 h)	Number of short stops (< 12 h) during transport	Categorical
Classes of short stops (<12 h)	Number of short stops (<12 h) during the journey without horses unloading categorized into two classes (between 0 and 2 short stops; more than 2 short stops)	Dichotomous
Long stops (>12 h)	Presence/absence of a stop during the journey with the horses unloaded from the vehicle, for feeding and watering (>12 h)	Dichotomous

Vehicle change	If horses were unloaded from one vehicle and loaded onto a different vehicle to continue the journey	Dichotomous
Stocking density (kg/m^2)	How many kg of live weight was distributed per m^2	Continuous
Horses per vehicle (n)	Number of horses on the vehicles	Continuous
Space allowance (m^2/animal)	m^2 available for each horse, net of partitions	Continuous
Space allowance classes	m^2 available for each horse, net of partitions, divided into classes (i.e., <1.75 m^2/animal, >1.75 m^2/animal)	Dichotomous
Load weight from CMR (kg)	Total weight of horses on a vehicle (from CMR)	Continuous
Load weight from TRACES (kg)	Total weight of horses on a vehicle (from TRACES)	Continuous
Animal in group (n)	Total number of horses in group	Continuous
Group size (n)	Number of horses per group (i.e., 2, 3 or 4)	Categorical
Tied	If the horses were tied in transit	Dichotomous
Direction of travel	The direction in which the horse travelled (i.e., 90° head right, 90° head left, free, oblique, forward, backward	Categorical
Season	Seasons in which horses travelled (i.e., Spring, Summer, Autumn, Winter)	Categorical
Arrival temperature (°C)	External environmental temperature measured through a weather station	Continuous
Fed during transport	Presence/absence of the feeding of horses during the journey	Dichotomous
Unloading duration (min)	From the lowering of the vehicle ramp until the last horse was unloaded measured with a stopwatch	Continuous
**Human-related variables**		
Driver's age (years)	Age of the vehicle driver	Continuous
Driver's experience (years)	Years of experience driving vehicles for the transport of horses	Continuous
Certificate of competence	Presence/absence of the certificate of competence	Dichotomous
The handler moves the animals in an arousing manner, inducing fear	The handler induces the horse/s to move with rapid movement of the body (i.e., arms or running, inducing fear)	Dichotomous
The handler moves the animals in a noisy manner, inducing fear	The handler induces the horse/s to move with loud sounds (i.e., shouting or by slamming, inducing fear)	Dichotomous
The handler hits the animals without reason	The handler hits the horse/s while it/there is/are moving or even if it/there is/are not able to move	Dichotomous
The handler moves the animals properly	The handler moves the horse/s without endangering the safety of the animal/s or their own	Dichotomous
The handler performs prohibited practices	The handler performs practices which are banned in Regulation EC 1/2005	Dichotomous
Beaten with a stick	The horse was beaten with a stick or rubber tube at unloading	Dichotomous
**Vehicle-related variables**		
Total vehicle dimension (m^2)	Internal dimensions of the vehicles	Continuous

Ramp height	Height from the highest point of the ramp to the ground	Continuous
Ramp slope	Compliance/non-compliance of the ramp slope with Regulation 1/2005	Dichotomous
Ramp flooring	Cover material for the unloading ramp (i.e., Non-slip knurled metal with foot battens; Corrugated metal; Rubber mat)	Categorical
Ramp side gates	Presence/absence of side gates on the ramp	Dichotomous
Ramp covering	Degree of coverage of the ramp with bedding material (i.e., None, Partial, Complete)	Dichotomous
Deck height	Height from the highest point of the ramp to the ground	Continuous
Vehicle floor bedding	Degree of coverage of the vehicle floor with bedding material (i.e., Partial, Complete)	Dichotomous
Vehicle bedding type	Bedding material for the vehicle floor (i.e., Straw, Shavings, Straw and shavings, Sand and shavings)	Categorical
Individual bays (n)	Number of bays housing one horse	Continuous
Individual bays dimension (m^2)	The width and the length of the individual bays	Continuous
Adjacent stalls	Presence of horses that cannot travel close together safely (mare/stallion or stallion/stallion)	Dichotomous
Drinkers (n)	Number of drinkers vs working drinkers on the vehicles	Continuous
Type of drinkers	Structure and characteristics of the drinkers (i.e., Portable, Fixed Bowls)	Dichotomous
Water tank	The filling level of the water tank (i.e., Partially Empty, Empty)	Dichotomous
Temperature monitoring system	Presence/absence of vehicle interior temperature monitoring system	Dichotomous
Cameras	Presence/absence of vehicle interior cameras	Dichotomous
Forced ventilation	Presence/absence of vehicle interior forced ventilation system	Dichotomous
Number of forced ventilators	Number of forced ventilators on the vehicles	Continuous
Lighting system for animals' orientation	Presence/absence of vehicle interior lighting system	Dichotomous
Lighting system for loading/unloading	Presence/absence of vehicle external lighting system	Dichotomous
Slot/steps	Presence/absence of empty space (>10 cm) between the vehicle's structures (vehicle/ramp /floor/doors)	Dichotomous
Blockage zone on the ramp	Presence/absence of holes or physical obstacles on the ramp which block the movements of the horses	Dichotomous
Sharp edges	Presence/absence of potentially dangerous sharp edges on vehicle structures	Dichotomous
Signs of diarrhoea in the vehicle	Presence/absence of signs of diarrhoea inside the vehicle	Dichotomous
**Horse-related variables**		
Horse ID	Horse identification based on passport's microchip number	Categorical
Horse's Age	Age of the individual horse	Continuous
Horse's Type	Categorization of morphology (i.e., light-breed horse, draft-breed horse)	Dichotomous

Horse's age*Horse's type	Interaction between horse's age (continuous) and horse's type (dichotomous)	Continuous
Halter	Presence/absence of the halter on the horse	Dichotomous
Dead on arrival	Number of dead horses per journey	Continuous
Non-ambulatory	Number of horses unable to independently assume and maintain a standing position per journey	Continuous
Lame horses	Number of horses which perform clear attempts to take the weight off one or more limbs per journey	Continuous
Injuries	Number of horses with injuries involving superficial or deeper tissues on the horse's body and potentially causing a loss of function per journey	Continuous
Injuries	Presence/absence of injuries (cuts and/or bruises on the animal's body)	Dichotomous
Injured tail	Presence/absence of injuries on the tail	Dichotomous
Injured head/neck	Presence/absence of injuries on the head and/or neck	Dichotomous
Lameness score	Scoring of the lameness severity from 0 to 5	Categorical
Clinical signs of colic	Horses showing signs of abdominal pain/discomfort	Dichotomous
Nasal discharge	Presence/absence of loss of material from the nose	Categorical
Type of nasal discharge	Which characteristics the nasal discharge has (i.e., color, consistency, smell)	Categorical
Lacrimal discharge	Presence/absence of loss of material from the eyes	Dichotomous
Other discharges	Presence/absence of loss of material from other orifices (i.e., penis, vagina etc…)	Categorical
Coughing	Presence/absence of horses'/'s cough attacks per journey or per single animal	Dichotomous
Heart Rate	Number of heartbeats in one minute	Continuous
Respiratory Rate	Number of respiratory acts in one minute	Continuous
Feces	Presence/absence of feces with an altered consistency	Dichotomous
Clinical signs of colic	Presence/absence of any sign of abdominal discomfort	Dichotomous
Other clinical signs	Presence/absence of any sign suggesting a loss of function in one of the main body systems	Dichotomous
Sweating	Number of horses showing signs of sweating on the bodies per journey/ Presence/absence of sweating signs on horse body	Continuous / Dichotomous
Coat	Presence of winter (long) or summer (short) hair on the animal	Dichotomous
Horse's Body Condition Score (BCS)	Number of horses per vehicle that present 0–1 or 5 score BCS	Continuous
Estimate weight	Estimated horse's weight based on assessors' evaluation	Continuous
Reluctance to move/unload	The horse showed unwillingness to move forward or move from its position during unloading per journey	Continuous
Slipping/Losing of balance	Number of horses showing uncontrolled limb movement due to slippery ground during unloading per journey	Continuous
Falling	Number of horses showing loss of balance with body touching the ground during unloading per journey	Continuous

Galloping and/or jumping	Number of horses galloping and/or jumping during unloading per journey	Continuous
Demeanour	State of the sensorium and reactivity to external stimuli (i.e., Alert/responsive, Alert/quiet, Lethargic, Depressed, Exhausted, Scared)	Categorical
**Lairage-related variables**		
Temperature	External environmental temperature measured through a weather station	Continuous
Relative humidity	External environmental relative humidity measured through a weather station	Continuous
Exposure	The exposure of the paddock/stabling to sun or shadow	Dichotomous
Bedding cleaning	The level of cleanliness of the bedding	Categorical
Number of horses per facility	Number of horses in stabling boxes (i.e., 1, 2, 3) or paddocks (i.e., 4-11, more frequently 6-7)	Categorical
Freedom of movement	Presence/absence of freedom of movement for the horse	Dichotomous
Declared level of animal handling	Declared level of taming at departure	Dichotomous
Broken/unbroken test (BUT)	Score 0-2 (x2) to the two phases (haltering and handling) of BUT	Categorical

Table S6. Associations between the predictive variables and the presence/absence of reluctance to move behavior at unloading in the 613 broken horses transported to two slaughterhouses in Italy. Associations are presented with Wald Test P-values.

Variable	Wald test P-value
Duration from Journey Log	<0.001
Unloading duration	<0.001
Driver's experience	0.500
Arrival temperature	0.532
Season	<0.001
Vehicle change	0.013
Long stops (>12h)	<0.001
Number of short stops classes	0.010
Fed during transport	0.101
Tied	<0.001
Ramp flooring	<0.001
Ramp side gates	0.764
Ramp lateral protections	0.118
Stocking density	0.008
The handler moves properly the horses	<0.001
Vehicle bedding type	<0.001
Type of drinkers	0.003
Horse's sex	0.485
Horse's age	0.005
Horse's BCS	0.111
Horse's type	0.018
Coat	0.313
Use of the stick	<0.001

Table S7. Associations between the predictive variables and the presence/absence of nasal discharge in the 613 broken horses transported to two slaughterhouses in Italy. Associations are presented with Wald Test P-values.

Variable	Wald test P-value
Duration from Journey Log	<0.001
Unloading duration	<0.001
Driver's experience	0.368
Arrival temperature	<0.001
Season	<0.001
Vehicle change	<0.001
Long stops (>12h)	0.984
Number of short stops classes	<0.001
Fed during transport	<0.001
Tied	<0.001
Ramp flooring	0.978
Ramp side gates	0.565
Ramp lateral protections	<0.001
Stocking density	<0.001
The handler moves properly the horses	0.983
Vehicle bedding type	<0.001
Type of drinkers	<0.001
Horse's sex	0.083
Horse's age	<0.001
Horse's BCS	<0.001
Horse's type	<0.001
Coat	0.014

Table S8. Associations between the predictive variables and the presence/absence of lacrimal discharge in the 613 broken horses transported to two slaughterhouses in Italy. Associations are presented with Wald Test p-values.

Variable	Wald test p-value
Duration from Journey Log	<0.001
Unloading duration	<0.001
Driver's experience	0.448
Arrival temperature	<0.001
Season	0.002
Vehicle change	<0.001
Long stops (>12h)	0.984
Number of short stops classes	<0.001
Fed during transport	<0.001
Tied	<0.001
Ramp flooring	0.287
Ramp side gates	0.986
Ramp lateral protections	<0.001
Stocking density	<0.001
The handler moves properly the horses	0.984
Vehicle bedding type	<0.001
Type of drinkers	<0.001
Horse's sex	<0.001
Horse's age	0.001
Horse's BCS	<0.001
Horse's type	<0.001
Coat	0.010
Nasal discharge	<0.001

Table S9. Associations between the predictive variables and the presence/absence of injuries and cuts in the 613 broken horses transported to two slaughterhouses in Italy. Associations are presented with Wald Test p-values.

Variable	Wald test p-value
Duration from Journey Log	<0.001
Unloading duration	<0.001
Driver's experience	0.106
Arrival temperature	0.027
Season	<0.001
Vehicle change	<0.001
Long stops (>12h)	<0.001
Number of short stops classes	<0.001
Fed during transport	0.009
Tied	<0.001
Ramp flooring	<0.001
Ramp side gates	0.478
Ramp lateral protections	0.228
Stocking density	<0.001
The handler moves properly the horses	<0.001
Vehicle bedding type	<0.001
Type of drinkers	0.036
Horse's sex	0.958
Horse's age	0.038
Horse's BCS	<0.001
Horse's type	<0.001
Coat	0.537

Table S10. Associations between the predictive variables and the presence/absence of injured tails in the 613 broken horses transported to two slaughterhouses in Italy. Associations are presented with Wald Test p-values.

Variable	Wald test p-value
Departure country	0.022
Duration from Journey Log	0.011
Unloading duration	0.006
Driver's experience	0.468
Arrival temperature	0.723
Season	0.366
Vehicle change	0.066
Long stops (>12h)	0.062
Number of short stops classes	0.054
Fed during transport	0.899
Tied	0.010
Ramp flooring	0.104
Ramp side gates	<0.001
Ramp lateral protections	0.988
Stocking density	0.005
The handler performs prohibited practices	0.068
Vehicle bedding type	<0.001
Type of drinkers	0.401
Horse's sex	0.313
Horse's age	0.005
Horse's BCS	0.002
Horse's type	0.224

Coat	0.508
Sweating	0.413
Beaten with a stick	0.586

Table S11. Associations between the predictive variables and the presence/absence of injured head/neck in the 613 broken horses transported to two slaughterhouses in Italy. Associations are presented with Wald Test p-values.

Variable	Wald test p-value
Departure country	<0.001
Duration from Journey Log	<0.001
Hours in transit	<0.001
Unloading duration	<0.001
Driver's experience	0.608
Arrival temperature	0.066
Season	<0.001
Vehicle change	0.003
Long stops (>12h)	<0.001
Number of short stops classes	0.003
Fed during transport	0.002
Tied	<0.001
Ramp flooring	<0.001
Ramp side gates	0.203
Ramp lateral protections	0.576
Stocking density	<0.001
The handler performs prohibited practices	<0.001
Vehicle bedding type	<0.001
Type of drinkers	0.014
Horse's sex	0.971
Horse's age	0.007
Horse's BCS	0.002
Horse's type	0.001
Coat	0.824
Sweating	0.413
Beaten with a stick	0.395
